# Development of mt-NADES Beads and Optimization for Efficient Extraction of Methylene Blue Using Response Surface Method

**DOI:** 10.1021/acsomega.6c00978

**Published:** 2026-05-26

**Authors:** Taiwo Bakare-Abidola, Seth K. Smerjac, Kyle Jorgensen, William J.A. Russell, Rahul Sampat Khandge, Rocío L. Pérez

**Affiliations:** Center for Advanced Materials Science (CAMS); Department of Biochemistry, Chemistry, and Physics, 7604 Georgia Southern University, Statesboro, Georgia 30458, United States

## Abstract

The escalating contamination of aquatic environments by synthetic dyes, particularly methylene blue (MB), represents a pressing environmental challenge, with far-reaching implications for ecosystem health and human welfare. Technologies such as adsorbents have been shown to be promising remediation technologies. Here, we report on the development of biopolymeric adsorbent beads by functionalizing alginate-chitosan matrices with menthol-thymol-based natural deep eutectic solvents (mt-NADES), demonstrating notable MB removal capacity. The strategic incorporation of NADES simultaneously enhances hydrophobic interactions and structural integrity, yielding superior dye adsorption toward the matrix. Response surface methodology (RSM) established an optimal operational parameter (275 ppm MB, 155 mg adsorbent, pH 10, 150 min contact time), achieving a maximum MB removal of 95.21%, remarkably concordant with the predicted value of 97.41%, showcasing strong model prediction. Kinetic analysis demonstrates pseudo-second-order adsorption behavior, while isotherm modeling confirms multilayer adsorption on heterogeneous active sites (Freundlich model), with thermodynamic parameters confirming spontaneous, endothermic adsorption characteristics. Remarkably, the mt-NADES beads demonstrated outstanding reusability, retaining >95% removal efficiency across six consecutive cycles, underscoring their practical viability. These NADES-functionalized biopolymeric beads provide a sustainable treatment approach for critical industrial wastewater needs while advancing the green chemistry principles.

## Graphical abstract

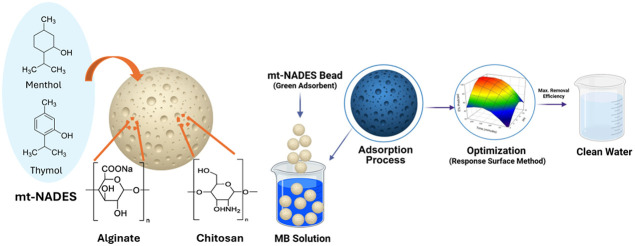

## 1 Introduction

The preservation of global water resources is critically threatened by industrial pollution, with synthetic dyes representing a persistent and recalcitrant class of contaminants.^1–3^ Annually, over 700,000 tons of dyes are produced globally, and a significant portion enters aquatic ecosystems through untreated or partially treated effluents from textile, leather, and printing industries.^4–6^ These complex aromatic compounds are designed for stability, leading to low biodegradability, long-term environmental persistence, and detrimental effects, including reduced light penetration, oxygen depletion, and acute toxicity to aquatic flora and fauna.^2,7^ The recalcitrance of dye molecules, coupled with the variable and complex chemistry of industrial wastewater, makes their complete removal a significant technical challenge driving urgent research into efficient, scalable, and economically viable remediation technologies.^8,9^

Methylene blue (MB), a heterocyclic, cationic thiazine dye, serves as a prevalent model pollutant due to its extensive use in coloring paper, dyeing cottons, wools,^10^ and in medical applications. Its presence in wastewater, even at low concentrations, is highly visible and concerning.^11,12^ While MB has medicinal uses at low doses, elevated concentrations pose substantial environmental and health risks, including toxicity to aquatic life and, in humans, potential symptoms ranging from nausea to more severe disorders.^13,14^ Increasingly stringent environmental regulations worldwide mandate the reduction of colorants in discharged effluents, creating a pressing need for effective decolorization methods that can meet these regulatory limits.^15^

Conventional wastewater treatment methods, such as coagulation-flocculation,^16,17^ advanced oxidation processes (AOPs),^18–20^ membrane filtration,^21,22^ and biological treatment,^23,24^ exhibit notable limitations; for e.g., coagulation generates large volumes of hazardous sludge,^25^ AOPs often incur high operational costs and can form toxic byproducts,^19^ membrane processes face fouling issues and high energy demands,^26^ and biological systems are generally inefficient against stable synthetic dyes.^20^ In contrast, adsorption has emerged as a highly promising alternative due to its simplicity of design, potential for adsorbent regeneration, applicability at various scales, and often superior efficiency, particularly for dilute solutions.^27–30^ The core of this technology lies in the development of high-performance, cost-effective, and sustainable adsorbents.

In the quest for sustainable adsorbents, biopolymers like alginate and chitosan have gained prominence. Alginate, an anionic polysaccharide extracted from brown seaweed, forms hydrogels in the presence of divalent cations (e.g., Ca^2+^), creating a porous three-dimensional “egg-box” matrix ideal for pollutant entrapment.^31^ Chitosan, a cationic polysaccharide derived from chitin, is notable for its high content of reactive amino (−NH_2_) and hydroxyl (−OH) groups, which act as effective coordination and electrostatic binding sites.^32–34^ Employing these biopolymers for the fabrication of composite beads capitalizes on their synergy, preventing excessive swelling, and providing a blend of anionic (carboxylate from alginate) and cationic (protonated amines from chitosan) functional groups for enhanced pollutant interaction.^35–38^

Recent advances in alginate-chitosan-based adsorbent design have increasingly focused on composites functionalizing to boost adsorption capacity, selectivity, and reusability. The incorporation of additives such as clay minerals,^39^ magnetic nanoparticles,^40^ activated carbon^41^ and ionic liquids into alginate-chitosan matrices has been widely explored to improve surface area, introduce new binding mechanisms, and facilitate separation.^42–44^ A particularly innovative approach is the integration of Deep Eutectic Solvents (DES) or their natural analogues (NADES) into solid supports.^45^ These solvents can act as multifunctional modifiers, imparting specific chemical functionalities (e.g., additional hydrogen-bond donors/acceptors, aromaticity) to the adsorbent surface, thereby tailoring its affinity for target contaminants.^46,47^ Choline chloride-based DES, often combined with urea, glycerol, or carboxylic acids, represent the most extensively studied class for material modification.^48^ These hydrophilic DES have been employed to modify clays,^49^ activated carbons,^50–52^ and biopolymers (e.g., alginate, chitosan),^53,54^ primarily enhancing surface functionality and introducing additional binding sites.^55^ Organic acid-based DES (e.g., citric acid, malic acid) have been explored for introducing carboxyl-rich functionalities, while metal salt-based DES offer potential for creating hybrid materials.^56^ More recently, hydrophobic DES, particularly those based on terpenes (menthol, thymol, camphor) and fatty acids, have gained attention as green alternatives for extraction and separation processes.^57^ These hydrophobic NADES offer unique properties, including low volatility, biodegradability, and the ability to engage in multiple interaction types (e.g., hydrogen bonding, π–π stacking, hydrophobic interactions) with aromatic compounds.^58^

NADES represent a class of green solvents typically formed from naturally occurring, biocompatible components like organic acids, sugars, and terpenes through hydrogen bonding (H-bonding) interactions.^59,60^ Their low toxicity, biodegradability, and tunable physicochemical properties align perfectly with green chemistry principles. Beyond their primary role as extraction media, NADES are increasingly being investigated as functional components in material science. When embedded within a solid matrix, they can introduce tailored interaction sites, such as H-bonding, π–π stacking, and hydrophobic interactions, significantly enhancing the adsorbent’s affinity for specific pollutants like synthetic dyes.^61,62^

Menthol-Thymol system, a hydrophobic NADES formed between the H-bond acceptor menthol and donor thymol, possesses structural properties featuring aromatic rings and strong H-bonding networks.^63^ Such structural characteristics are highly conducive to interacting with aromatic and amine moieties of MB through a combination of π–π interactions, hydrophobic effects, and potential H-bonding, which have been identified as dominant adsorption mechanisms for methylene blue on functionalized adsorbents.^64^ Therefore, the selection of a menthol–thymol NADES (mt-NADES) for this study is strategically motivated by its potential to exploit these synergistic interaction pathways.

A review of the current literature reveals a distinct knowledge gap. While studies on alginate-chitosan beads^65^ and, separately, on NADES applications in extraction are abundant, the strategic functionalization of a dual biopolymeric bead system with a hydrophobic, terpene-based NADES for enhanced dye adsorption has been only sparsely investigated.^66–68^ Recently, Luna-Díaz et al., incorporated hydrophobic terpene-based NADES (menthol:camphor) into PVP-alginate beads for the extraction of parabens, achieving enhanced signal.^69^ In another study, an amino acid-based DES (adipic acid:l-cysteine) was developed in cellulose-alginate beads for phthalate extraction, demonstrating remarkable reusability.^70^ In contrast, studies on DES-modified adsorbents have focused on hydrophilic systems, with Martínez-Rico et al. demonstrating that chitosan beads modified with choline chloride:urea DES exhibit enhanced adsorption of azo dyes.^53,71^ Despite these advances, a knowledge gap still remains.^69^

There has also been no reported systematic study that has employed a response surface methodology (RSM) approach to simultaneously optimize the development and application of biopolymer NADES beads for the sequestration of dye, specifically MB.^72,73^ This understanding is scientifically significant, as it advances the rational design of next-generation green adsorbents and is practically important for developing more efficient, sustainable, and optimized treatment protocols for dye-contaminated water.

In this study, we postulate that embedding mt-NADES within an alginate-chitosan biopolymeric network yields a unique composite adsorbent with a heterogeneous and synergistically active surface chemistry. The integration of mt-NADES is expected to couple the electrostatic and hydrophilic interactions inherent to the biopolymer matrix with the complementary hydrophobic and π–π interaction capabilities of the NADES, thereby enhancing both the adsorption capacity and removal of MB relative to unmodified beads. To systematically evaluate this hypothesis, key operational parameters, including solution pH, adsorbent dosage, initial dye concentration, contact time, and temperature, were investigated. Furthermore, response surface methodology (RSM), employing Central Composite Design, was adopted in place of conventional one-factor-at-a-time approaches to enable efficient experimental design, capture factor interactions, and identify optimal adsorption conditions.

## 2 Experimental Section

### 2.1 Synthesis and Characterization of mt-NADES

The mt-NADES was synthesized following a previously reported method with minor modifications (Scheme S1).^74^ Briefly, thymol and DL-menthol were combined in an equimolar ratio (1:1) in a clean, dry glass vial. The mixture was heated at 65 °C in an oil bath with continuous magnetic stirring (300 rpm) for 30 min until a homogeneous, transparent liquid was formed. The resulting mt-NADES remained in liquid state (Figure 1a) upon cooling to room temperature (25 °C). The synthesized mt-NADES was stored in a sealed amber vial at room temperature until further use. The mt-NADES was characterized by FT-IR, ^1^H NMR, ^13^C NMR, and LCMS-ESI spectroscopy to verify its structural confirmation and purity.

**Figure 1:**
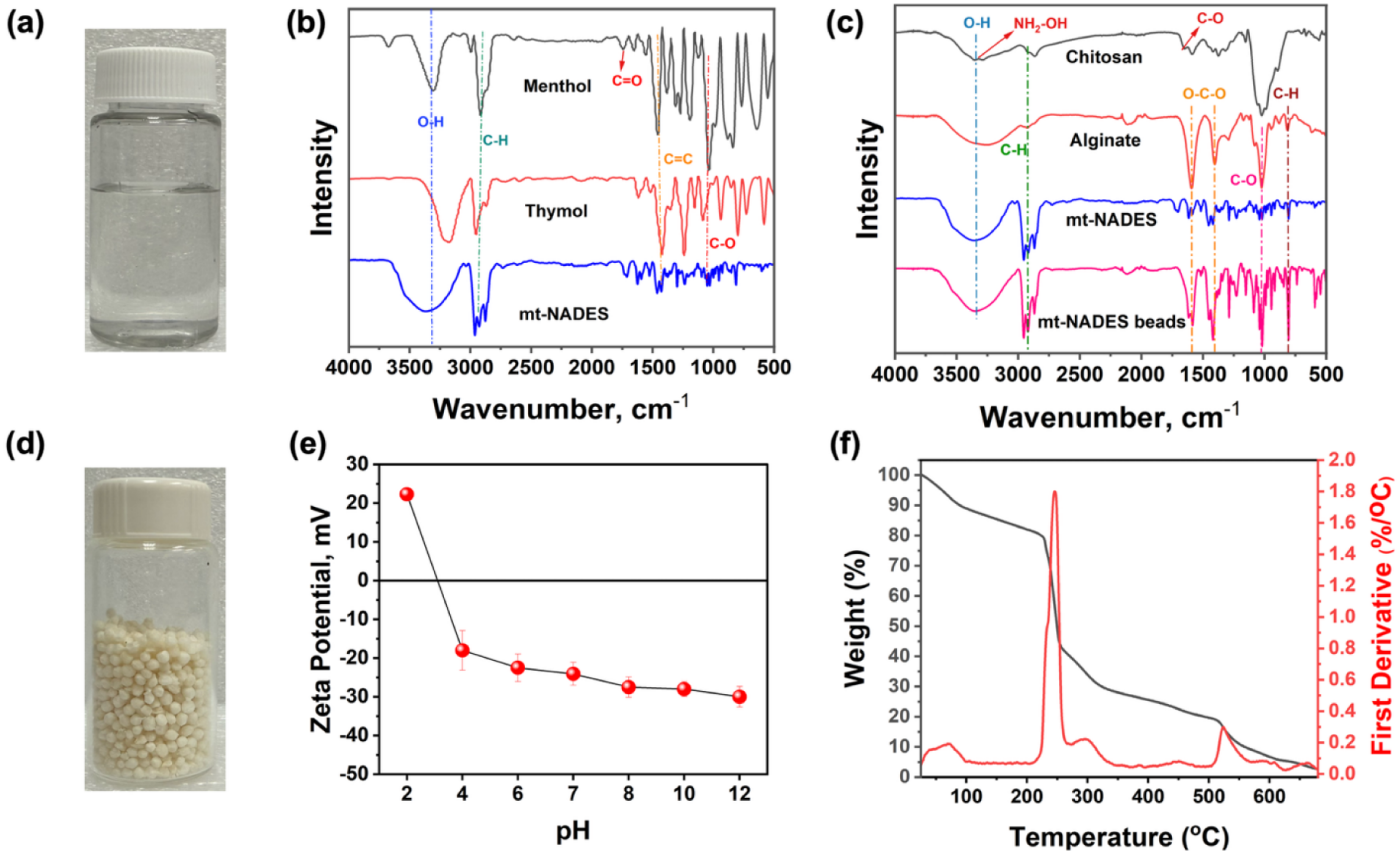
(a) Pictorial of mt-NADES; (b) FT-IR spectra of menthol, thymol, and mt-NADES; (c) FT-IR spectra of chitosan, alginate, mt-NADES, and mt-NADES beads; (d) pictorial of mt-NADES beads; (e) zeta potential of mt-NADES beads at different pH; (f) thermogravimetric curve of mt-NADES beads.

### 2.2 Synthesis and Characterization of mt-NADES Beads

Scheme 1 provides an overview of the fabrication process of mt-NADES beads. Briefly, aqueous solutions of alginate and chitosan (each at 1.2% w/v) were combined to form a homogeneous biopolymer matrix. A volume of mt-NADES sufficient to reach a 1% loading in the biopolymer matrix was then incorporated into the solution under continuous stirring, allowing uniform dispersion of the hydrophobic NADES within the polymer matrix. After complete homogenization, the mixture was loaded into a syringe and dispensed using a syringe pump at a controlled flow rate of 1.5–2.0 mL/min to ensure consistent droplet size and bead morphology. The droplets were introduced into a 2% w/v CaCl_2_ solution, where rapid ionic cross-linking of alginate with Ca^2+^ induced immediate cross-linking of the biopolymers, allowing the bead formation. The resulting mt-NADES beads were allowed to be cured in the CaCl_2_ solution overnight. Subsequently, the beads were collected by filtration, frozen, and lyophilized to remove residual water while preserving their internal microstructure.

**Scheme 1:**
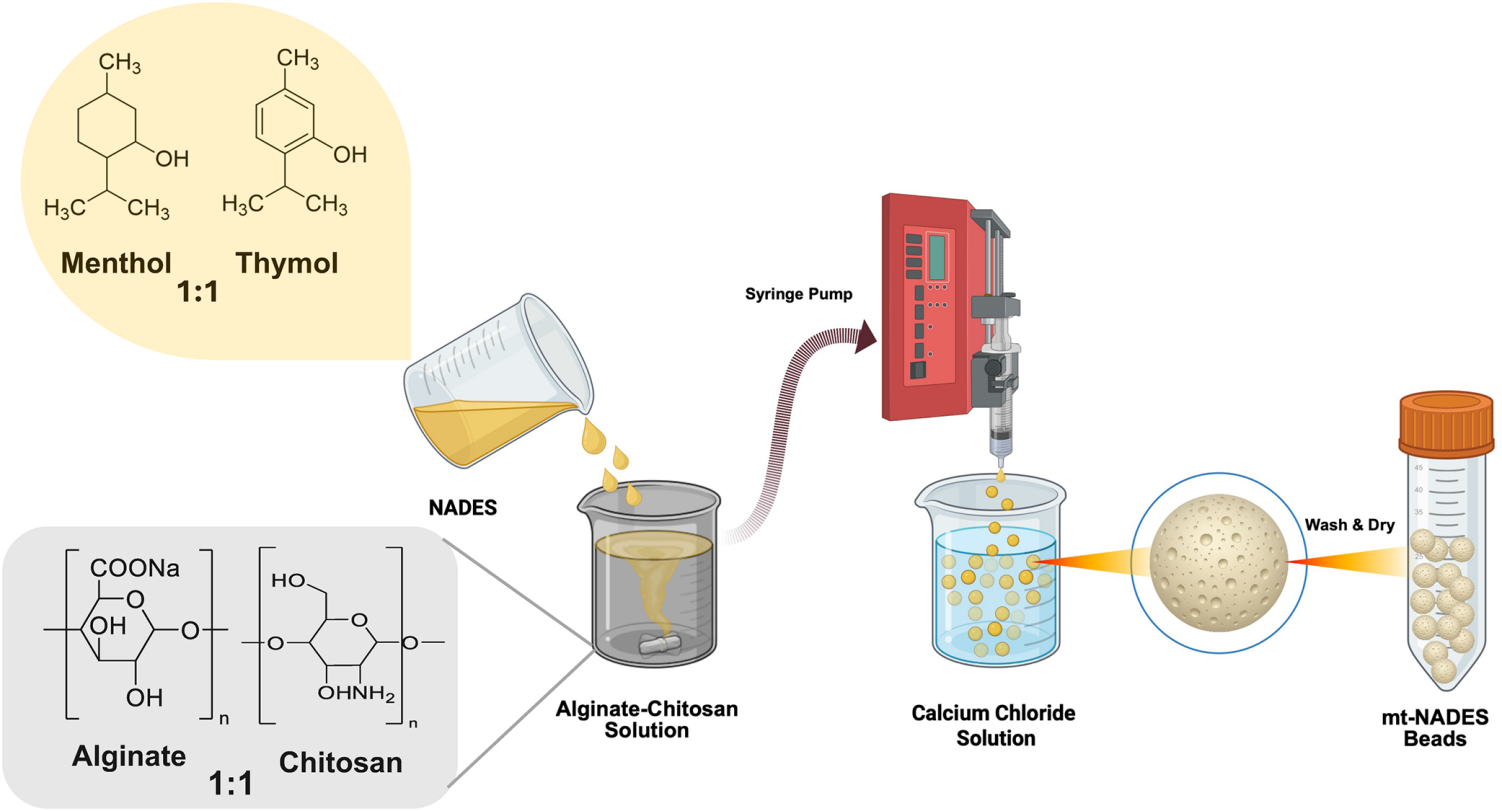
Illustration of the Synthesis of mt-NADES Beads

### 2.3 Extraction Batch Procedure

An MB stock solution was prepared by accurately weighing 10 mg of dye, placing it in a 10 mL volumetric flask, and taking it up to volume with Milli-Q water. The working solutions were prepared daily by dilution of the stock solution. For each extraction experiment, the desired mass of mt-NADES beads was weighed into vials, and the MB solution was added at a predetermined concentration and adjusted to the working pH. Then, the vials were placed on the shaker at 200 rpm for the corresponding extraction time. The concentration of MB was monitored by spectrophotometric absorbance before and after the extraction procedures. Removal efficiency was subsequently calculated using eq 1. Furthermore, eq 2 was used to calculate the adsorbent capacity after 24 h.

### 2.4 Experimental Design, Optimization, and Model Evaluation

A two-level Central Composite Design (CCD) was used for optimization of four independent variables with the objective of maximizing the removal percentage of MB (dependent variable) using the synthesized mt-NADES beads. In this work, four independent variables, namely pH, bead’s mass (m, mg), MB concentration (MB, ppm), and time (t, min) at the three levels (−1, 0, 1) as seen in Table 1, were employed to create the CCD with a total of 26 experiments, and one response removal percentage (R%) was studied.

**Table 1:** Factors and Values of the Evaluated Levels in the CCD

Evaluated parameters	Low level	Central level	High level
pH	2	7	12
Mass (mg)	20	110	200
MB (ppm)	50	275	500
Time (min)	15	127.5	240

Following completion of the 26 experimental runs, the data were analyzed using analysis of variance (ANOVA). Model significance was evaluated using Fisher’s test to assess the contribution of the regression, lack of fit (LOF), and the coefficient of determination (R^2^). The experimental design and statistical analyses were carried out using JMP Pro 17. The resulting data were used to develop an empirical mathematical model and corresponding response surface methodology (RSM) to identify the experimental conditions that maximize MB removal efficiency (R%). The validity of the RSM model was assessed by comparing experimentally measured MB removal efficiencies with the values predicted by the model across various conditions, including the optimized conditions. All experiments were conducted in triplicate to ensure reproducibility and accuracy.

### 2.5 Adsorption Isotherm, Kinetic, and Thermodynamics Study

Adsorption isotherm studies were performed at pH 10 and ambient temperature with MB concentrations ranging from 50 to 500 ppm until equilibrium conditions were achieved. The equilibrium data obtained were subsequently fitted to the Freundlich, Langmuir, and Temkin isotherm models for analysis.

Kinetic experiments were carried out by adding 50 mg of beads to 10 mL of an MB solution (15 ppm, pH 10). The suspension was maintained under continuous agitation, and the MB concentration was monitored at various time points between 15 and 240 min. The collected data were subsequently modeled using widely applied pseudo-first-order (PFO) and pseudo-second-order (PSO) kinetic equations.

Thermodynamic analyses were performed at varying temperatures (304–328 K) to investigate the impact of temperature on the adsorption efficiency of MB onto the beads. A volume of 10 mL of MB solution (15 ppm, pH 10) was combined with 0.1 g of beads and agitated in a shaking bath maintained at the designated temperatures. Equilibrium adsorption capacities were measured after a 24 h contact time at each temperature. The thermodynamic feasibility and nature of the adsorption process were evaluated by calculating the Gibbs free energy (Δ*G*), enthalpy change (Δ*H*), and entropy change (Δ*S*). These parameters were determined from the slope and intercept of a ln *K* versus 1/T plot, offering valuable insight into the thermodynamic behavior of the system.

### 2.6 Reusability and Recyclability

The recyclability and regeneration performance of the beads were assessed through repeated adsorption–desorption cycles reported elsewhere.^75^ In brief, 100 mg of beads was contacted with 10 mL of MB solution (15 ppm, pH 10). The adsorbed dye was removed by vigorous agitation in the presence of different desorbing agents chosen by literature review, such as sodium hydroxide (NaOH), hydrochloric acid (HCl), a 0.5 M NaCl water solution, and a 0.5 M NaCl 50:50% v/v (EtOH/H_2_O) solution. Each regeneration medium was put in contact with the used beads placed in the shaker for a period of 3 h. Afterward, the beads were separated by filtration and reused in a fresh MB solution for two cycles to evaluate the best desorption medium. From the results obtained (Table S8), the optimal desorbing agent was chosen (0.5 M NaCl 50:50% v/v (EtOH/H_2_O), and the reusability of the beads was further evaluated by six consecutive cycles using the best desorption agent. All obtained data were analyzed in accordance with eqs 1 and 2.

### 2.7 Data Analysis and Error Functions

The adsorption behavior of MB on beads was investigated utilizing the formula:

1 $$R\%=\frac{\left(C_{0}-C_{t}\right)}{C_{0}}\times 100$$

where *C*_0_ represents the initial concentration, while *C*_*t*_ is the concentration after the subsequent time periods.

Adsorbent capacity measured at 24 h and over various time intervals was determined in accordance with the following equation:

2 $$q_{e,t}=\frac{\left(C_{0}-C_{e}\right)}{m}\times V$$

where *C*_*e*_ is the equilibrated concentration (mg·L^–1^), *C*_0_ is the initial concentration (mg·L^–1^), *m* is the mass of the beads (g), and *V* is the volume of the solution (L) in which the adsorption was carried out.

To assess the agreement between experimental data and theoretical predictions, a set of error functions was employed in this study. The chi-square function (χ^2^), the sum of squared errors (SSE), and the mean squared error (MSE) serve as standard measures for assessing model performance for some of the error functions taken into consideration. The predicted values (*q*_*e*,*cal*_) are compared with that of the experiment (*q*_*e*,*exp*_), and the best fit model is selected. The error determined by each function characterizes error differently, providing complete information on model experiments and providing a sound assessment of adsorption processes.

3 $$\chi^{2}=\overset{n}{\underset{i=1}{\sum }}\frac{{\left(q_{e,exp}-q_{e,cal}\right)}^{2}}{q_{e,cal}}$$

4 $$MSE=\frac{1}{N_{exp}}\sum {\left(q_{e,exp}-q_{e,cal}\right)}^{2}$$

5 $$SSE=\sum {\left(q_{e,exp}-q_{e,cal}\right)}^{2}$$

### 2.8 Determination of Point of Zero Charge (pH_PZC_)

The pH at the point of zero charge (pH_PZC_) of the mt-NADES beads was determined using the pH drift method following established protocols with minor modifications.^75^ In brief, a series of 50 mL aliquots of 0.01 M NaCl solution were prepared in 100 mL Erlenmeyer flasks to maintain constant ionic strength. The initial pH (pH_i_) of each solution was adjusted to values between 2.0 and 12.0 using 0.1 M HCl or 0.1 M NaOH solutions, and the exact initial pH of each solution was recorded using a calibrated pH meter (precision ± 0.01). Accurately weighed 0.1 g of mt-NADES beads were added to each vial bottle, and the vial bottles were sealed and shaken in an orbital shaker at 250 rpm for 24 h at 25 °C to reach equilibrium. After 24 h, the final pH (pH_f_) of each solution was measured.

The pH drift was calculated using the following equation:

6 $$\Delta pH=pH_{i}-pH_{f}$$

The ΔpH values were plotted against the corresponding initial pH (pH_i_), and the pH_PZC_ was determined as the x-intercept of the resulting curve, i.e., the pH at which ΔpH = 0, corresponding to the condition where pH_i_ = pH_f_. All measurements were performed in triplicate, and the average pH_PZC_ value is reported with standard deviation. The pH drift method is based on the principle that at pH < pH_PZC_, the surface is positively charged and adsorbs OH^–^ ions (or releases H^+^), causing the solution pH to increase toward pH_PZC_. Conversely, at pH > pH_PZC_, the negatively charged surface adsorbs H^+^ ions, causing pH to decrease toward pH_PZC_.^76^

## 3 Results and Discussions

### 3.1 Synthesis and Characterization of mt-NADES

Herein, menthol and thymol, two naturally occurring organic monoterpenoids, were mixed in a 1:1 molar ratio to synthesize mt-NADES (Figure 1a). The successful formation of mt-NADES was confirmed using a suite of analytical techniques. First, FT-IR spectroscopy revealed a notable shift in the O–H stretching vibration to 3316 cm^–1^ in mt-NADES compared to 3307 cm^–1^ and 3185 cm^–1^ for menthol and thymol (Figure 1b), indicating the presence of strong intermolecular H-bonding, consistent with the formation of a eutectic system. ^1^H NMR spectroscopy further confirmed structural interactions within the eutectic mixture. Characteristic chemical shift changes, including downfield shifts of hydroxyl protons and subtle alterations in the aliphatic regions (Figure S1), demonstrate molecular interactions between menthol and thymol, confirming the formation of a thermodynamically stable eutectic mixture. The ^13^C NMR spectrum of the synthesized mt-NADES (Figure S2) provides direct evidence of successful eutectic formation. While signals corresponding to both menthol and thymol are present, key differences from the neat precursors are observable. Notably, several carbon resonances exhibit slight but consistent chemical shift changes (Δδ ∼ 0.1–0.5 ppm) compared to the spectra of pure compounds. More importantly, a general broadening of the signals is evident. These phenomena are diagnostic of strong intermolecular interactions, specifically the formation of a H-bond network between the hydroxyl group of menthol and the phenolic −OH of thymol. This network alters the local electronic environment of the constituent carbons and reduces molecular tumbling rates, leading to the observed peak broadening. The spectrum confirms that the mt-NADES is not a simple physical mixture but a distinct, homogeneous liquid phase stabilized by intermolecular forces.

The mt-NADES synthesis was further confirmed through ESI-MS presented in Table S1, exhibiting the expected [M + H]^+^ ions for thymol (*m*/*z* ∼ 151) and menthol (*m*/*z* ∼ 156), with no additional peaks or adduct species detected. These results further indicated that eutectic formation arises from noncovalent H-bonding interactions^77^ rather than any covalent chemical transformation of the constituents.

To evaluate the physicochemical suitability of the synthesized NADES, key properties including melting point, density, and viscosity were measured for the pure components (menthol and thymol) and their eutectic mixture. As summarized in Table S2, the mt-NADES showed a pronounced melting-point depression (∼10 °C) relative to the individual components (menthol: 42–45 °C; thymol: 49–51 °C), a defining thermodynamic signature of deep eutectic formation driven by strong cooperative H-bond interactions. The measured density (0.91 to 0.97 g/cm^3^ falls within the expected range for hydrophobic NADES, indicating structural compactness consistent with efficient packing of the constituent molecules.

This hydrophobicity, which is crucial for the intended application, arises from the intrinsically nonpolar nature of its components.^68,78^ Furthermore, the viscosity (27.5 to 53 mPa·s, depending on temperature and shear conditions) is well-suited for applications requiring rapid mass transfer, such as liquid–liquid extraction, encapsulation of hydrophobic active compounds, and solvent-mediated catalytic processes.^66,79^ Collectively, these physicochemical characteristics underscore the stability, processability, and application versatility of the mt-NADES system.

### 3.2 Synthesis and Characterizations of mt-NADES Beads

FT-IR spectroscopy was employed to confirm the successful preparation of the composite mt-NADES beads and to investigate the molecular interactions, particularly H-bonding, between the biopolymer matrix (alginate and chitosan) and the mt-NADES. The spectra of the individual component’s chitosan, alginate, mt-NADES, and mt-NADES beads are presented in Figure 1c. Chitosan spectrum did exhibit its characteristic O–H and NH_2_–OH spectra at 3400 cm^–1^ along with a weak C–H stretching at 2900 cm^–1^ with a saccharide C–O stretching between 1000 and 1150 cm^–1^. Alginate is seen to show the similar O–H band with a pronounced carboxylate asymmetric and symmetric stretching at ∼1600 cm^–1^ and ∼1410 cm^–1^ and a strong vibrational C–O peak at 1030 cm^–1^.^80^ These characteristic peaks from chitosan, alginate, and mt-NADES are well-defined in the mt-NADES beads. For instance, the broad O–H/N–H region (3000–3500 cm^–1^) undergoes clear broadening and shifting, indicating extensive new H-bonding interactions between the biopolymers and mt-NADES. Concurrently, the shift in alginate carboxylate bands (1400–1600 cm^–1^) in position and intensity evidences ionic cross-linking and polymer–NADES interactions, while the retention of C–H and C–O signals in the 900–1300 cm^–1^ fingerprint region, including the characteristic C–H stretching and bending vibrations in the parent material as well as the mt-NADES beads spectra, confirms the stable entrapment of the mt-NADES within the matrix, unequivocally verifying the successful fabrication of the composite beads.^81,82^ To determine the charge of the mt-NADES beads, zeta potential (ZP) analysis was obtained (Figure 1e). At highly acidic pH (e.g., pH 2.0), the ZP is strongly positive, reaching a maximum of +22 mV, which could be attributed to the protonation of the chitosan amine groups (−NH_3_^+^). Conversely, as the pH increases, the ZP rapidly becomes negative, stabilizing around −30 mV ± 0.6 in the basic range (pH 10–12).^83^ This negative charge is primarily due to the deprotonation of the alginate carboxyl groups (−COO^–^). The highly negative potential observed across the neutral to basic pH range is indicative of a robust, stable colloidal system and suggests that the beads would be highly effective for the adsorption or encapsulation of cationic compounds such as MB, which is the target pollution in this study. This also highlights a significant advantage for applications requiring pH-responsive materials. Furthermore, to precisely define the net surface charge of the mt-NADES beads (i.e., surface charge is neutral at certain pH), the point of zero charge (pH_PZC_) was determined using the pH drift method. From the analysis, the pH_PZC_ of the mt-NADES beads was determined to be approximately at pH 8.2. This value is highly significant for interpreting the adsorption mechanism of the beads toward the cationic MB dye. For pH < 8.2, the surface of the adsorbent carries a net positive charge because of the protonation of the amino (−NH_2_) groups present in the chitosan component (Figure S3). Under these conditions, the surface would primarily favor the adsorption of anionic species. For pH > 8.2, the surface acquires a net negative charge due to the deprotonation of the carboxyl (−COOH) groups in the alginate component. This negative surface charge creates a strong electrostatic driving force for the adsorption of cationic species.

TGA was performed to understand the thermal stability and the decomposition stages of the synthesized mt-NADES bead (Figure 1f). The TGA plot confirms that the composite beads exhibit remarkable thermal stability, maintaining structural integrity up to approximately 200 °C, with the minimal initial weight loss observed below this temperature being solely attributable to the evaporation of physically adsorbed moisture.^84^ The primary thermal event, corresponding to the degradation of the organic components (the mt-NADES and the biopolymeric matrix), is characterized by a sharp, well-defined decomposition step with an onset temperature of approximately 275 °C. The Derivative Thermogravimetric (DTG) curve highlights this major event with a distinct, narrow peak centered between 270 and 300 °C.^85^ The sharpness and singular nature of this peak are critical, as they strongly suggest a high degree of structural homogeneity within the bead composition, where the encapsulated NADES and the biopolymer matrix degrade in a highly cooperative and synchronized manner. This high decomposition temperature, significantly exceeding typical operational limits for environmental and catalytic applications, confirms the high thermal robustness of the synthesized beads, positioning them as a highly stable and durable material.

FE-SEM was conducted to elucidate the surface morphology of the dehydrated mt-NADES beads. The micrographs presented in Figure 2a reveal a predominantly spherical morphology with an average diameter of 1.8 ± 0.3 mm, indicating uniform bead formation across the batch. Higher-magnification imaging (Figure 2b–f) shows that the bead surface is characterized by a distinct flower-like topology, consisting of irregular folds, ridges, and shallow depressions, which together give rise to a rough, textured surface morphology for the mt-NADES beads.^86,87^ The observed texture is consistent with reported morphologies of porous alginate-chitosan composites, where surface roughness has shown indication of increased surface area.^88^ Such morphologies have also been correlated with improved adsorption performance in biopolymer-based adsorbents.^86,87^

**Figure 2:**
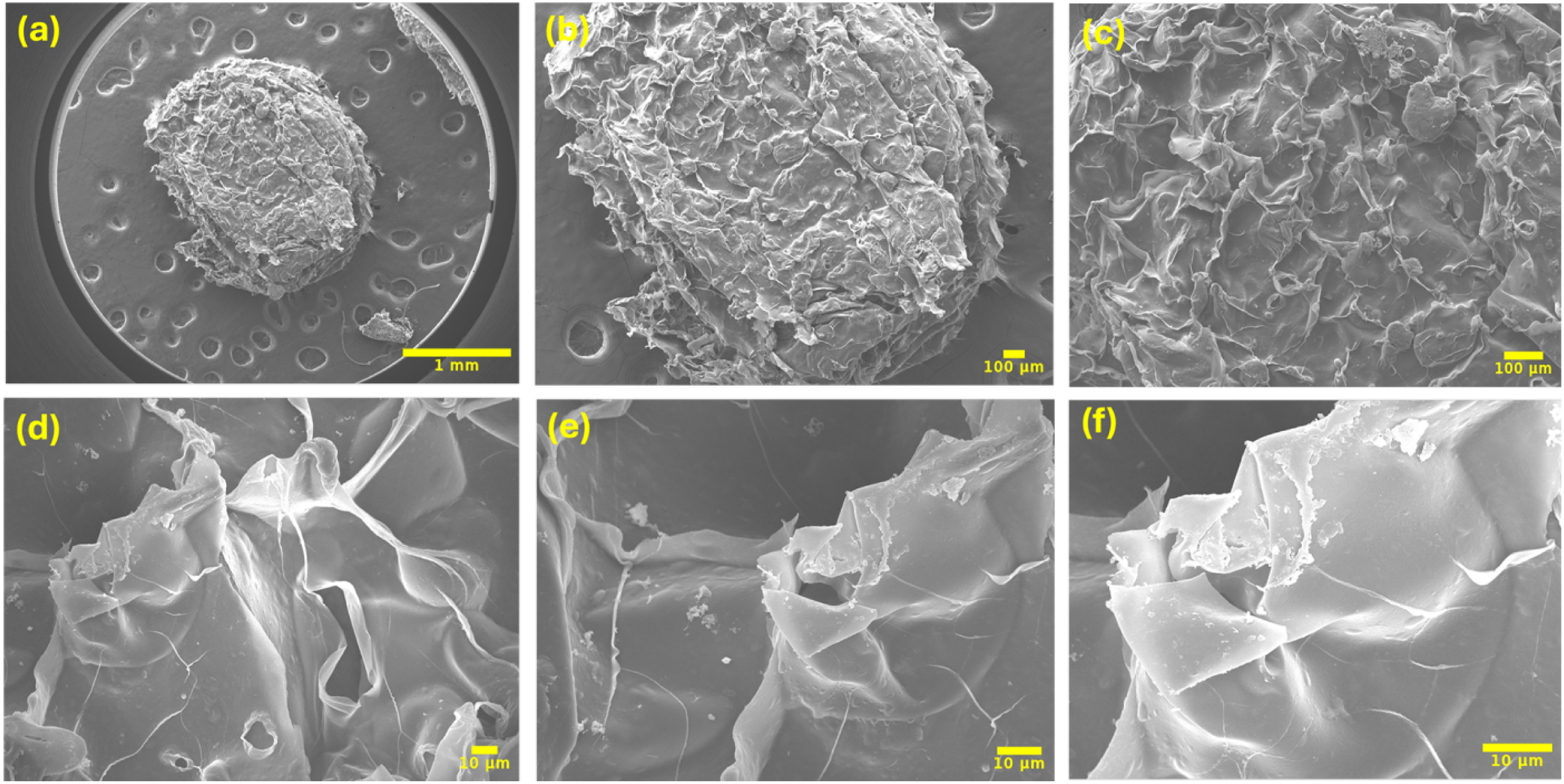
FE-SEM pictograms of mt-NADES beads at six different magnification scales: (a) 25×, (b) 50×, (c) 100×, (d) 600×, (e) 1000×, and (f) 1500×.

### 3.3 Model Evaluation and Experimental Design

The removal efficiency (R%) of MB was systematically evaluated using a CCD to identify the optimal experimental conditions yielding the maximum percentage removal. As summarized in Table S3, the CCD generated a total of 26 experiments based on 4 independent variables, each evaluated at three levels (Figure 3a). Analysis of these results indicates that the highest R% was achieved on experiment 9, corresponding to the simultaneous application of the highest evaluated levels of bead mass and extraction time, indicating a strong positive contribution of these parameters to dye removal. Additionally, experiments 6, 8, 15, 16, and 18 also exhibited comparably high R%, ranging between 90 and 97%.

**Figure 3:**
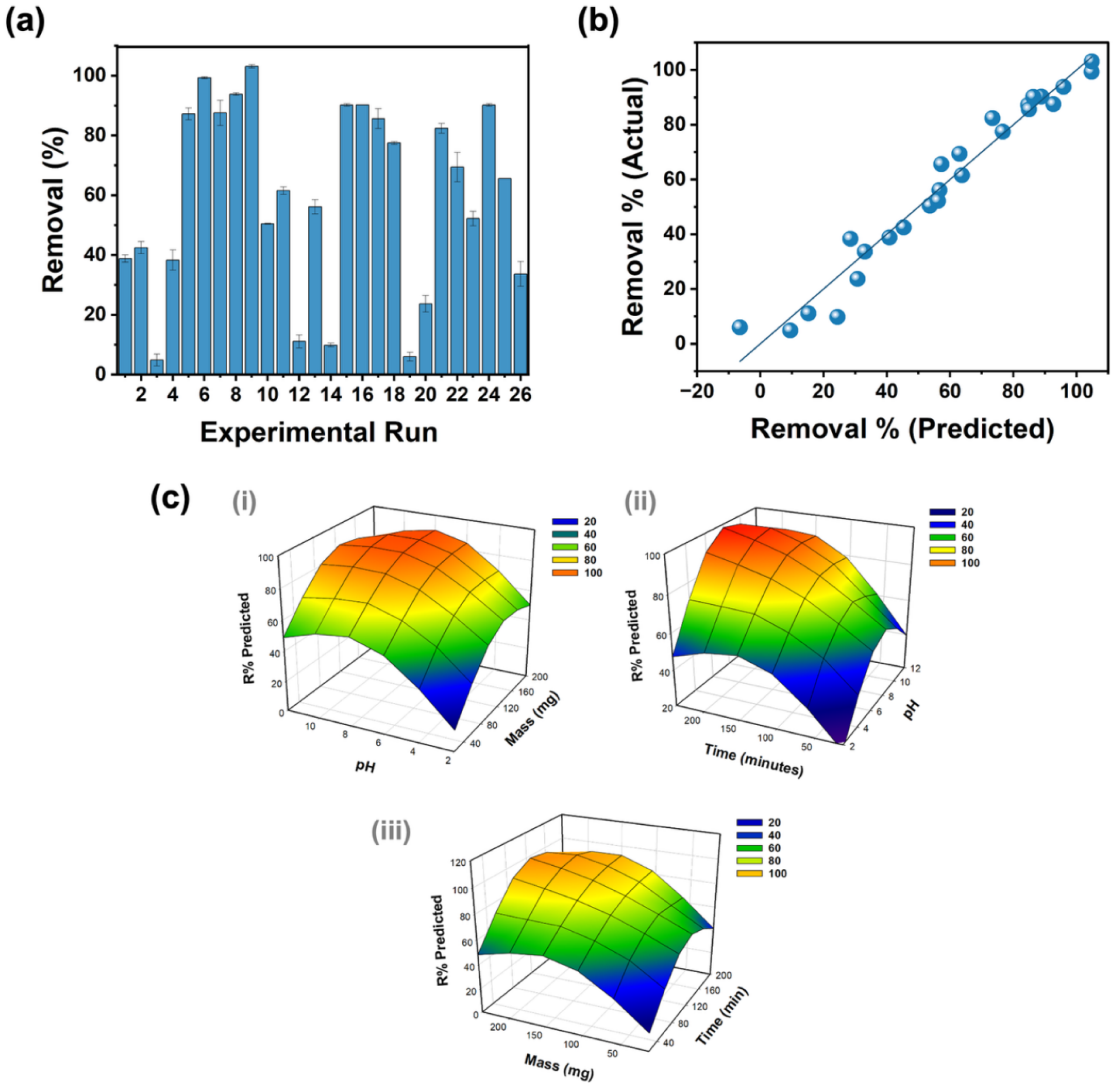
(a) Removal percentage (R%) of MB from aqueous solution at each experimental run; (b) predicted versus actual R% values; (c) three-dimensional response surface plots for the combined effect of (i) pH and mass, (ii) time and pH, and (iii) mass and time on the R% response.

However, despite the presence of these high-performing conditions, the data sets as a whole do not exhibit an immediately discernible trend, a consequence of the inherent structure of the CCD in which multiple variables are intentionally modified simultaneously. This multifactor interplay underscores the necessity of subsequent statistical modeling to resolve the individual and interactive effects of the evaluated parameters on MB removal.

The results obtained with the CCD were subjected to statistical validation through an evaluation of the studentized residuals for each experimental run, as presented in Figure S4, to identify potential outliers.^68^ All residuals remained well within the predefined control limits, confirming the absence of anomalous data points and the reliability of the experimental measurement. To further access model performance, ANOVA was conducted to assess the accuracy of the model generated through the RSM for the optimization of R% of MB (Table 2). The achieved model exhibited strong statistical significance (*p* < 0.0001) as well as a nonsignificant lack of fit (*p* = 0.2013), showcasing superior agreement between the model and the experimental data. Variables with *p*-values of <0.05, specifically bead mass, pH, initial MB concentration, and contact time, were identified as statistically significant factors governing the removal efficiency. The model demonstrated a high coefficient of determination (R^2^ = 0.958), confirming that 95.8% of the observed variance in MB removal was captured by the predictive model, underscoring its robustness and predictive reliability.

**Table 2:** ANOVA for the CCD for R% of MB from Aqueous Samples^a^

Source	SS	DF	MS	*F*-value	*p*-value
Model	23752.01	14	1696.57	20.96	<0.0001
Mass	4139.44	1	4139.44	42.95	<0.0001
pH	5022.32	1	5022.32	52.11	<0.0001
[MB]	453.49	1	453.49	4.71	0.0529
Time	7669.16	1	7669.16	79.57	<0.0001
Mass × pH	154.44	1	154.44	1.60	0.2317
Mass × [MB]	360.04	1	360.04	3.74	0.0794
pH × [MB]	203.49	1	203.49	2.11	0.1741
Mass × Time	41.16	1	41.16	0.43	0.5268
pH × Time	461.88	1	461.88	4.79	0.0510
[MB] × Time	53.37	1	53.37	0.55	0.4724
Mass × Mass	451.51	1	451.51	4.68	0.0533
pH × pH	696.99	1	696.99	7.23	0.0211
[MB] × [MB]	20.97	1	20.97	0.22	0.6500
Time × Time	233.05	1	233.05	2.42	0.1482
Lack of Fit	1052.9	10	105.29	14.57	0.2013
Error	890.41	11	80.95		
Pure Error	7.22	1	7.22		
Cor Total	24642.41	25			

a R^2^ = 0.9583; R^2^ adj = 0.9052; SS = sum of squares, DF = degree of freedom, and MS = mean square.

The predictive capability of the response surface model was further validated by comparing the experimentally measured removal efficiencies with the corresponding model-predicted values, as illustrated in Figure 3b. The strong alignment between these data sets confirms the model’s robustness, with the linear regression yielding an R^2^ value of 0.9583, demonstrating that the predictive output reliably reproduces the experimental observations.

Building on these results, the combined analysis of the CCD data set and the ANOVA enabled the development of a statistically rigorous mathematical model that incorporates all statistically significant variables influencing MB removal. The equation developed is a polynomial equation that allows the prediction of R% at different values of the independent variables (Eq 7).

7 $$R\%=85.44+15.17\times \frac{\left(m-110\right)}{90}+16.70\times \frac{\left(pH-7\right)}{5}+20.64\frac{\left(t-127.5\right)}{112.5}+{[\frac{\left(pH-7\right)}{5}]}^{2}\times -16.50$$

Three-dimensional response surface plots were generated to quantitatively elucidate the interactive effects of the process variables on dye removal efficiency (R%), and the resulting surfaces reveal distinct synergistic behaviors across some of the evaluates variable (Figure 3c). In Figure 3c–i, a pronounced cooperative effect between pH and adsorbent dose is observed, where concurrent increases in both parameters yield a progressive increment in R%, indicating that higher alkalinity enhances electrostatic attraction while increasing sorbent mass amplifies the availability of active binding sites. Comparable synergistic trends are evident in Figure 3c-ii and c-iii, where the combined influence of adsorbent mass with contact time, and contact time with pH, respectively, results in steep gradients in the response surface, demonstrating that extended interaction time facilitates intraparticle diffusion and surface complexation processes that are further amplified under alkaline conditions. The RSM model predicted optimal operational conditions at a bead mass of 155 mg, contact time of 150 min, initial dye concentration of 275 ppm, and a solution pH of 10 (Table S4), under which the theoretical removal efficiency reached 97.41%. Experimental validation under these optimized parameters for both unmodified (alginate-chitosan) and modified (mt-NADES) beads produced an actual removal efficiency of 55.91 ± 0.4% and 95.21 ± 0.4% (Table S4). In addition, the model prediction for the modified beads closely aligns with the theoretical R%, thereby confirming both the robustness of the RSM optimization and the high extraction capability of the mt-NADES beads for MB in aqueous media.

### 3.4 MB Isotherm Study

To elucidate the equilibrium dynamics and surface interaction mechanisms governing (MB) adsorption, a comprehensive isotherm study was conducted by varying the initial MB concentrations (50–500 ppm) at a constant pH of 10. The resulting equilibrium data, presented in Figure 4 and Table S5, were rigorously evaluated using the Freundlich, Langmuir, and Temkin isotherm models. The analysis demonstrates that the Freundlich model provides the most physically meaningful representation of the adsorption process, yielding a superior correlation coefficient (R^2^ = 0.9717). The calculated Freundlich intensity parameter (n) of 1.62 is greater than 1, indicating a favorable and cooperative adsorption process on a heterogeneous surface. This strong fit is a critical finding, as it signifies that the uptake of MB onto the mt-NADES beads is not a simple monolayer event but a more complex process governed by a nonuniform distribution of binding site energies and likely involving multilayer adsorption.

**Figure 4:**
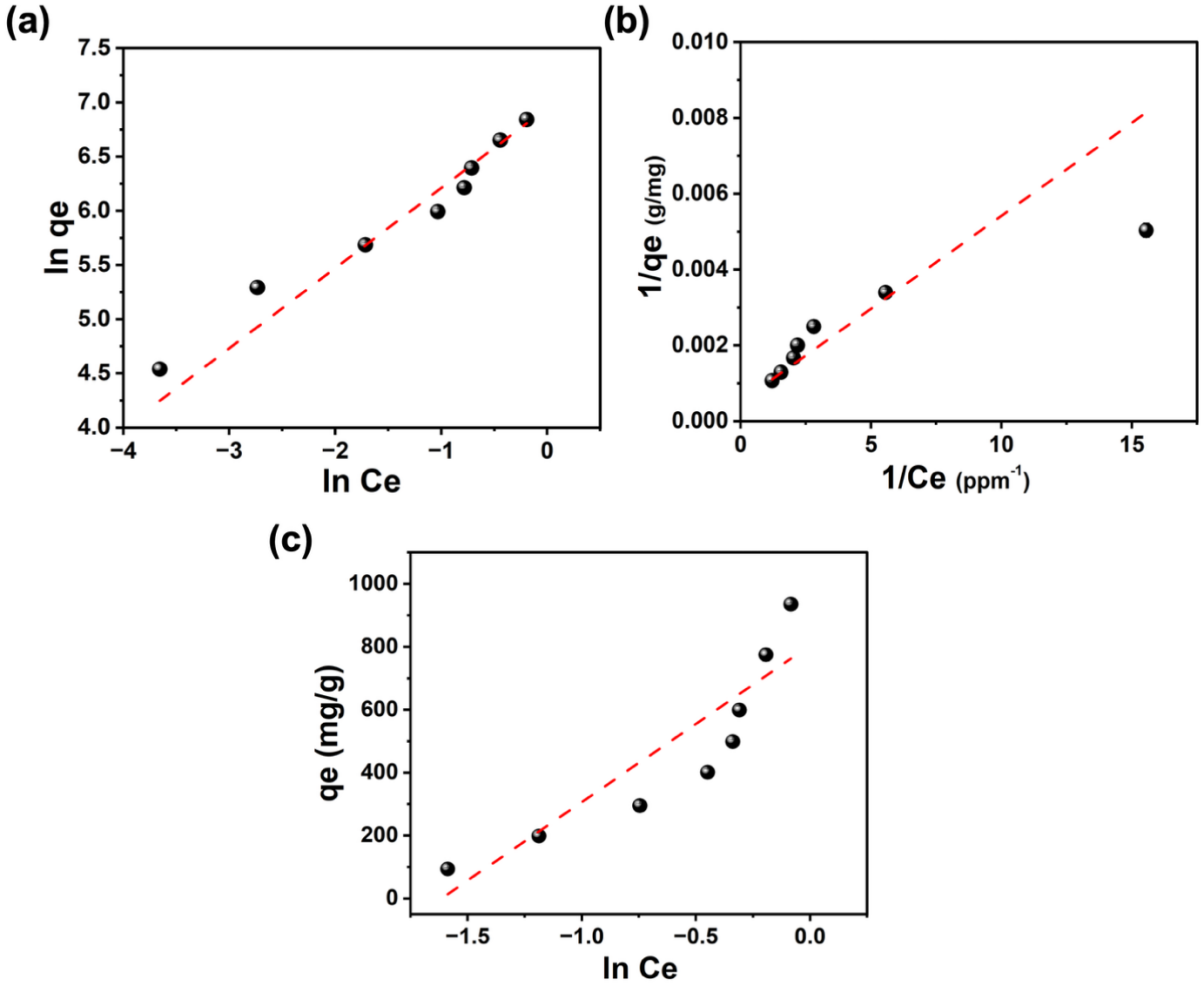
Adsorption isotherms of MB onto mt-NADES beads: (a) Freundlich, (b) Langmuir, and (c) Temkin models.

While the Langmuir model predicted a significant theoretical maximum monolayer adsorption capacity (*q*_max_) of 769.23 mg/g, its subordinate correlation coefficient (R^2^ = 0.8931) and higher chi-squared value (χ^2^ = 5.88 × 10^–3^) compared to Freundlich suggest it is less representative of the true adsorption mechanism. The Temkin model yielded the lowest correlation (R^2^ = 0.8118), further reinforcing that a simple model assuming uniform binding energies is inadequate. Therefore, despite the high theoretical capacity suggested by the Langmuir model, the collective evidence robustly points to the Freundlich isotherm as the best descriptor. This confirms that the mechanism for adsorption is a heterogeneous, multilayer process, highlighting the complex and energetically diverse nature of the active sites on the mt-NADES bead surface.

### 3.5 Kinetic Modeling

The adsorption kinetics of MB onto the synthesized mt-NADES beads were tested to reveal the underlying adsorption mechanism as well as to determine the rate-limiting mechanism (Figure 5). The PFO model, which supposes that the rate of filling the adsorption sites with material is directly proportional to the amount of unfilled sites, is represented as:

8 $$\ln\left(q_{e}-q_{t}\right)=\ln q_{e}-k_{1}t$$

**Figure 5:**
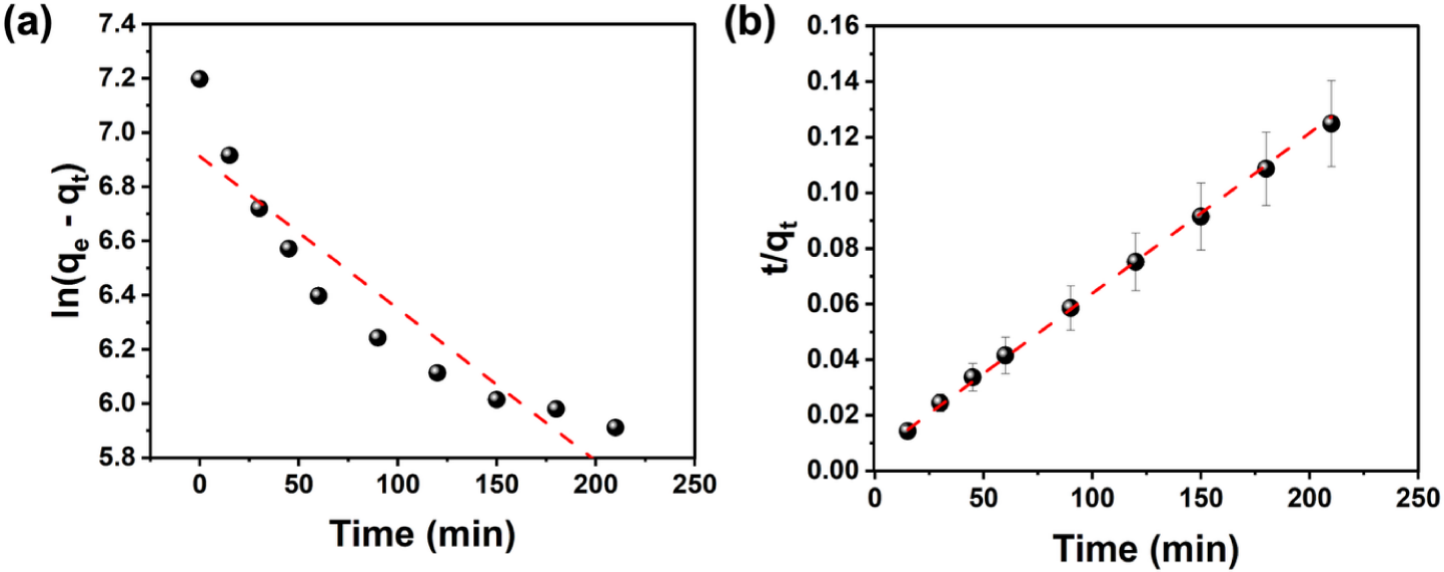
Linear representation of the experimentally measured data points of the measured data along with the fitted curves of both (a) PFO and (b) PSO models.

In contrast, the PSO model assumes chemisorption as the rate-controlling step and is represented in its linear form by the equation:

9 $$\frac{t}{q_{t}}=\frac{1}{k_{2}q_{e}^{2}}+\frac{t}{q_{e}}$$

In the context of adsorption kinetics, *q_t_* denotes the adsorptive capacity (mg g^–1^) measured at a particular time (*t*), whereas *q_e_* represents the equilibrium capacity. The constants *k*_1_ and *k*_2_ are, respectively, the rate parameters of the pseudo-first-order (PFO) and pseudo-second-order (PSO) kinetic models.

The results, summarized in Table S6, unequivocally showcase the dominance of the PSO model in explaining the adsorption kinetics. The PSO model yielded an outstanding correlation coefficient (R^2^ = 0.9971) and, critically, the calculated equilibrium capacity (*q_e_*,_*calc*_ = 181.81 mg/g) showed strong agreement with the experimental equilibrium capacity (*q_e_*,_*exp*_). In stark contrast, the PFO model provided a significantly poorer fit (R^2^ = 0.8753) and predicted a *q*_*e*_ value that was physically inconsistent with the experimental data. This strong correlation to the PSO model is a critical finding, as it provides compelling evidence that the rate-limiting step is chemisorption. This indicates that the adsorption mechanism is not governed by simple mass diffusion but rather by the formation of strong chemical interactions, likely through the H-bonds and π–π stacking interactions within the MB molecules and active functional groups on the bead surface. The high correlation of the PSO model, combined with the failure of the PFO model, confirms that physisorption plays a negligible role. This chemisorption-dominant mechanism underscores the highly specific and robust interactions engineered into the mt-NADES adsorbent, positioning it as a highly efficient and powerful material for environmental remediation applications where strong, stable binding of contaminants is paramount.

### 3.6 Thermodynamic Study of the Adsorption

The thermodynamic parameters governing MB adsorption onto mt-NADES beads were evaluated to elucidate the influence of temperature on adsorption efficiency. Experimental values of the equilibrium concentration (*C*_*e*_) and adsorption capacity (*q*_*e*_) were used to calculate key thermodynamic parameters, including enthalpy change (Δ*H*), entropy change (Δ*S*), and Gibbs free energy change (Δ*G*), using the following equations:

10 $$K_{eq}=\frac{q_{e}}{C_{e}}$$

11 ΔG=−RTln⁡K

12 ΔG=ΔH−TΔS

13 $$\ln K=\frac{\Delta S}{R}-\frac{\Delta H}{RT}$$

Whereas *K*_*eq*_ denotes the equilibrium constant of the adsorption process across different temperatures, and it is determined from the adsorption capacity (*q*_*e*_) in conjunction with the equilibrium concentration of MB (*C*_*e*_). Table S7 shows the thermodynamic parameters (Δ*G*) calculated from the experimental data. The entropies (Δ*S*) and enthalpies (Δ*H*) were calculated from a Van’t Hoff plot (Figure 6).

**Figure 6:**
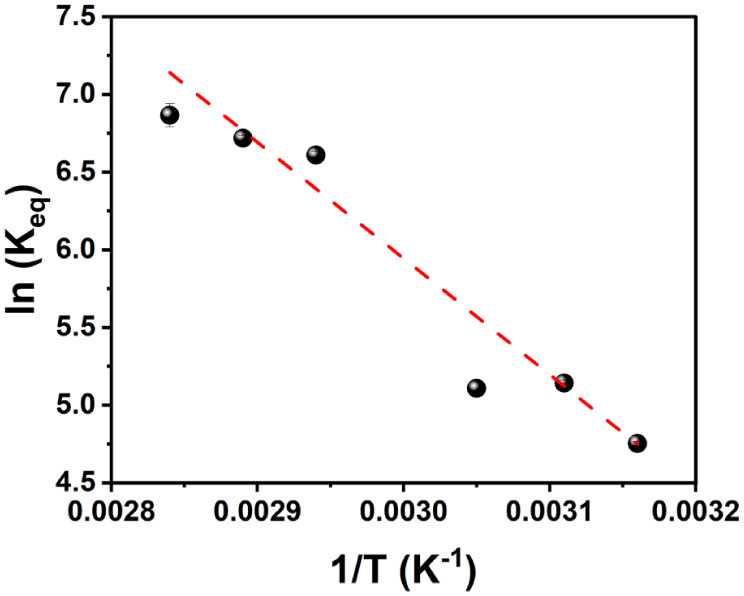
Van’t Hoff plot for the MB adsorption onto the mt-NADES beads.

The consistently negative values of Δ*G* across the entire temperature range, from −12.50 kJ/mol at 310 K to a more negative −19.52 kJ/mol at 352 K, unequivocally confirm that the adsorption of MB onto the beads is a spontaneous and thermodynamically favorable process. The positive value for the change in enthalpy (Δ*H* = +125.32 kJ/mol), derived from the slope of the Van’t Hoff plot, provides definitive evidence that the adsorption mechanism is endothermic in nature. This is a critical finding, as it explains the observed increase in adsorption capacity at higher temperatures. The endothermic character suggests that energy input is required to overcome activation barriers, facilitating the diffusion of MB molecules into the bead’s porous structure and their interaction with active sites. This is further supported by the increasingly favorable (more negative) Δ*G* values as temperature rises. Furthermore, the negative entropy change (Δ*S* = −174.97 J/mol·K) indicates a decrease in the randomness at the solid–liquid interface upon adsorption, as the MB molecules assume a more ordered, constrained state on the bead surface. Collectively, these thermodynamic results paint a clear picture of a spontaneous, endothermic, and entropy-driven process, highlighting the significant role of temperature in enhancing the efficiency of the mt-NADES beads for MB remediation.

### 3.7 Adsorption Mechanism

The adsorption of MB onto the mt-NADES beads may be attributed to multiple physicochemical interactions occurring on the surface and within the porous matrix of the adsorbent (Figure 7). The hybrid bead structure formed by the ionic cross-linking of alginate and chitosan provides a robust functional scaffold in active binding sites, as confirmed by the presence of abundant hydroxyl (−OH), amino (−NH_2_), and carboxyl (−COOH) groups in the FTIR spectra (Figure 1c). The strategic incorporation of the mt-NADES is a critical design element that significantly enhances the adsorption efficiency by introducing additional H-bonding sites and augmenting the overall hydrophobic character of the adsorbent surface. For these reasons, we hypothesized that the adsorption mechanism would be primarily governed by four concurrent interactions: (1) Electrostatic Attraction: At the optimized operational pH, the zeta potential analysis confirms that the bead surface is rendered highly negative due to the deprotonation of the −COOH and −OH groups (Figure 1e). This highly anionic surface promotes a powerful electrostatic attraction toward the cationic MB molecules, which could be one of the dominant driving forces for the initial rapid uptake of the dye. Further analysis using FTIR (Figure S5) found that the C=O stretching band observed in the fresh beads at approximately 1630–1650 cm^–1^ exhibited a change in intensity along with a slight shift toward higher wavenumber after adsorption. This behavior suggests a change in the local electronic environment of the carbonyl-containing groups and is consistent with the involvement of these sites in electrostatic interactions with MB molecules;^71^ (2) π–π Stacking Interactions: The presence of the aromatic structures (C=C) from the thymol component of the mt-NADES is pivotal for the adsorption of MB. These phenolic rings enable π–π stacking interactions with the aromatic rings of the MB dye,^89^ leading to enhanced and stabilized retention of the dye molecules on the adsorbent surface. A detailed FTIR analysis further supported the presence of π–π stacking interactions by showcasing that the aromatic C=C band around ∼1550 cm^–1^ had a slight reduction in intensity and narrowing of the band after adsorption, which may indicate the contribution of π–π interactions, likely between the aromatic moieties of thymol in the mt-NADES phase and the aromatic structure of methylene blue.^90^ This mechanism could also be a key contributor to the selectivity and capacity observed in the system; (3) H-bonding: The presence of numerous hydroxyl (O–H) and amino groups (N–H), as seen in the FTIR (Figure 1c), contributed by the alginate and chitosan biopolymers, along with the hydroxyl groups from the mt-NADES, serves as strong H-bond donors and acceptors.^38^ Further analysis of FTIR (Figure S5) confirmed that the broad O–H/N–H stretching region at approximately 3200–3500 cm^–1^ became broader and slightly shifted after dye uptake, suggesting the possible involvement of hydrogen-bonding interactions between functional groups on the adsorbent surface and methylene blue.^53^ The numerous hydroxyl (O–H) and amino groups (N–H) as seen in the FTIR (Figure 1c), contributed by the alginate and chitosan biopolymers, along with the hydroxyl groups from the mt-NADES, serve as strong H-bond donors and acceptors. H-bonding between these functional groups and nitrogen heteroatoms of the MB molecules would further stabilize the adsorption process, contributing to the overall binding energy and the potential for repetitive remediation cycles;^36^ (4) Physical Adsorption and Pore Diffusion: The porous, three-dimensional structure of the hydrogel beads facilitates the physical uptake of dye molecules. van der Waals forces contribute to nonspecific physical adsorption,^91^ while intraparticle pore diffusion ensures the migration of MB molecules deep within the internal structure of the beads, maximizing the utilization of all available binding sites.^92^

**Figure 7:**
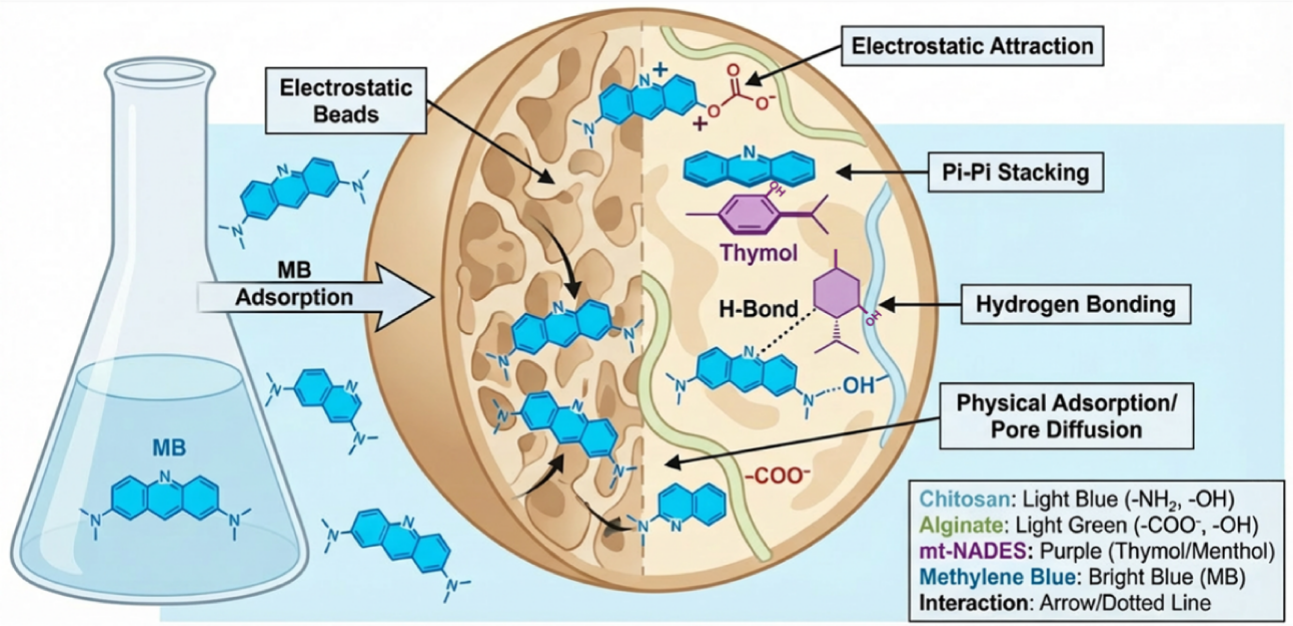
Illustration for the MB dye removal mechanism with the use of mt-NADES composite beads.

Thermodynamic investigations further support this multimechanistic model, revealing that the adsorption process is spontaneous (−Δ*G*) and endothermic (+Δ*H*). The endothermic nature of this adsorption process indicates that higher temperatures favor the migration of MB molecules to the adsorbent surface, reflecting a process that involves both physisorption and chemisorption components. While detailed characterization would further reinforce the proposed mechanism, the successful combination of alginate, chitosan, and mt-NADES thus creates a robust, multifunctional adsorbent where the synergistic effects of electrostatic, π–π, and H-bonding interactions collectively account for the high removal efficiency and structural integrity potential demonstrated by the composite beads.

### 3.8 Reusability

To evaluate the potential of the developed mt-NADES beads for real-world applicability and long-term stability, their reusability was investigated as an initial step. Accordingly, the adsorption–desorption performance was systematically assessed over six consecutive cycles under the as-prepared solution conditions. FE-SEM images (Figure 8a–d) of the beads after the sixth cycle reveal that the characteristic flower-like topology observed in Figure 2 is well preserved, even at the highest magnification, indicating minimal surface defects. Moreover, the overall spherical geometry of the beads remains intact after repeated use (Figure 8e). The adsorption performance exhibited high stability, with the removal efficiency showing only a marginal decline from 96.7% in the first cycle to 95.9% in the sixth cycle (Figure 8f). The consistently high removal efficiency, averaging approximately 97.5% across all cycles, highlights the robust structural integrity and chemical stability of the mt-NADES beads.

**Figure 8:**
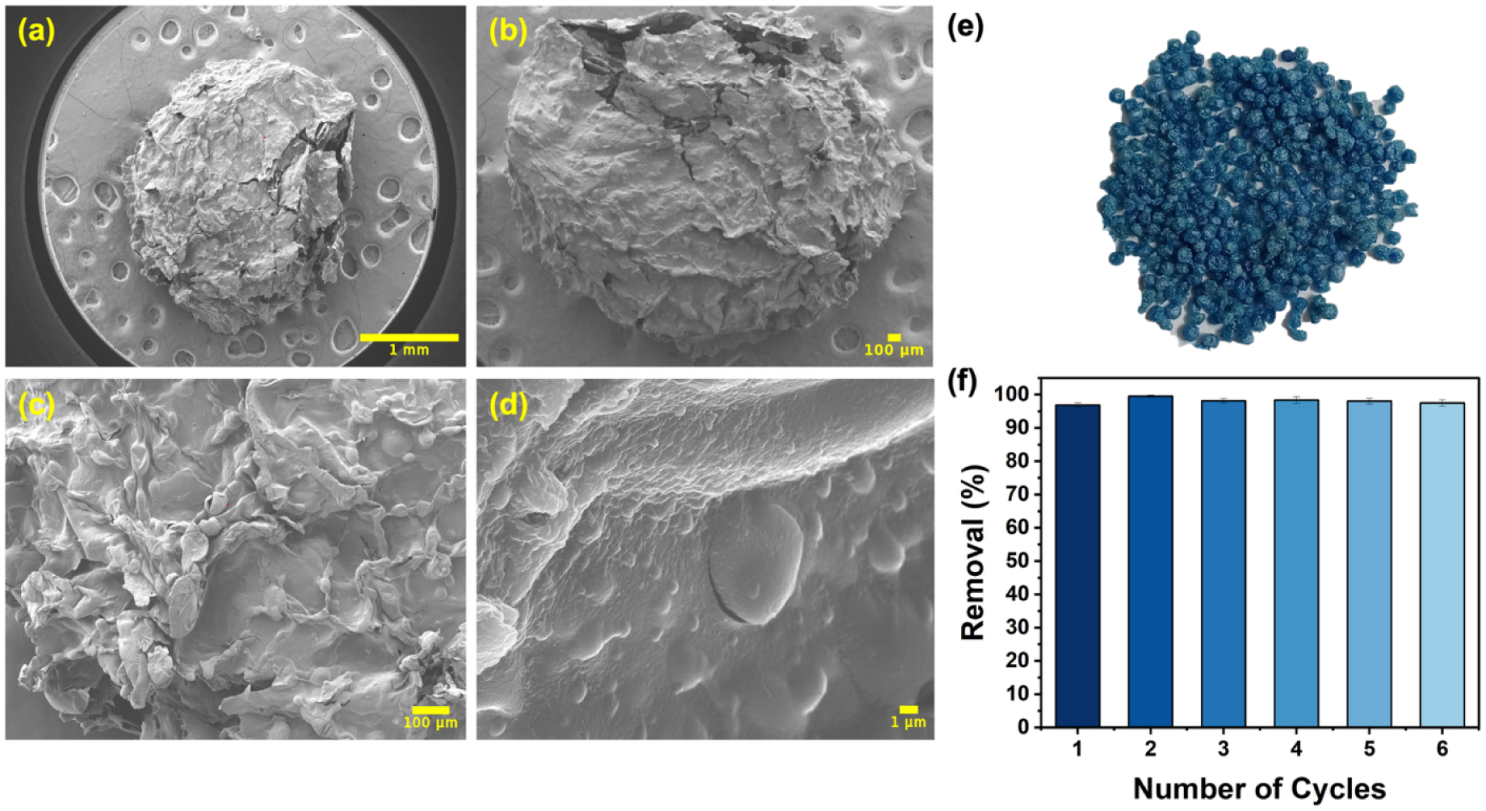
(a–d) Surface morphology of the reused mt-NADES beads using FE-SEM; (e) pictorial of mt-NADES beads; and (f) removal efficiency of MB under different cycles.

Notably, the adsorbent’s performance did not degrade even after prolonged use, maintaining its high efficiency throughout the repeated cycles. The slight increase in removal to 99% observed in the second cycle may suggest an activation phenomenon where the initial regeneration process potentially exposes additional binding sites. This outstanding and consistent performance through multiple regeneration cycles confirms that the mt-NADES beads are not only highly efficient but also robust and economically feasible. While long-term stability analysis is needed to confirm applicability under realistic wastewater conditions, the observed ability to maintain a high performance across reusability cycles demonstrates that these beads will be a superior, sustainable adsorbent with great potential for long-term scalable treatment systems.

### 3.9 Comparative Assessment with Previously Reported Adsorbents

To clearly position the novelty of this work, the adsorption performance of the prepared mt-NADES beads was benchmarked against previously reported biopolymer-based adsorbents for methylene blue (MB) removal (Table 3). The mt-NADES beads exhibit a comparatively high adsorption capacity (*q_e_* = 769.23 mg g^–1^), outperforming several conventional systems such as EDTA–chitosan/alginate beads (660.76 mg g^–1^)^93^ and acryloyl starch/carboxymethyl cellulose hydrogels (483.5 mg g^–1^),^94^ while significantly exceeding more traditional composites such as clay–alginate beads (109.9 mg g^–1^)^95^ and corncob cellulose-based hydrogels (33.0 mg g^–1^).^96^ Although certain graphene oxide-based systems demonstrate higher adsorption capacities (up to 1131 mg g^–1^),^97,98^ these materials often rely on nanocarbon additives associated with higher synthesis costs and potential environmental and health concerns.

**Table 3:** Performance Comparison of mt-NADES Beads with Other Reported Adsorbents for MB Removal

Adsorbent	MB Initial Conc. (ppm)	Adsorbent Dosage (g/L)	pH	Temp. (K)	Time (min)	Removal Efficiency (R%)	Adsorption Capacity *q*_*e*_ (mg/g)	References
EDTA-chitosan/alginate porous composite beads	100	1.0	6.0	298	1440	—	660.76	^93^
Graphene oxide-chitosan composite granules	100–500	1.0	7.0	303	240	—	951.35	^98^
Xanthan gum-chitosan/graphene oxide hydrogel	100–500	0.5	7.0	298	30	—	1131.0	^97^
Clay-alginate beads	25–200	2.0	7.0	298	180	>90%	109.9	^95^
Cellulose-clay-sodium alginate composite	25–150	0.5	7.0	303	60	>90%	—	^99^
Corncob cellulose-based hydrogel	74.5	2.22	7.0	303	80.4	98.25%	33.0	^96^
Acryloyl starch/carboxymethyl cellulose hydrogel	100	0.2	7.0	—	30	96.7%	483.5	^94^
Activated carbon from coffee grounds	15–50	15.0	7.0	298	45	99.15%	—	^100^
Marigold-derived activated carbon	20	2.5	4.0	—	—	>90%	14.02	^101^
mt-NADES/alginate-chitosan Beads	100–275	0.155	10	-	150	95.21	769.23	**This work**

In contrast, the key novelty of this work lies in the integration of natural deep eutectic solvents (NADESs) within a biopolymer matrix, which provides a synergistic enhancement in adsorption performance through tunable intermolecular interactions (e.g., hydrogen bonding and electrostatic interactions) without the need for engineered nanomaterials. This approach enables a balance between high adsorption capacity, material sustainability, and environmental compatibility, an aspect that remains underexplored in the current literature.

Overall, these results demonstrate that mt-NADES-functionalized biopolymer beads offer a competitive and sustainable alternative to cationic dye removal. It should be noted, however, that direct comparisons are made with caution because of variations in experimental conditions (e.g., initial concentration, pH, dosage, and temperature) across different studies.

## 4 Conclusion

This study presents the successful synthesis and evaluation of newly developed alginate-chitosan biopolymer beads by incorporating a menthol-thymol Natural Deep Eutectic Solvent (mt-NADES). The incorporation of mt-NADES enhanced the structural integrity, as well as the negative charge, of the biopolymer composite beads. They also showed higher thermal stability at around 200 °C. A process optimization study using Response Surface Methodology (RSM) and a Central Composite Design (CCD) enabled systematic exploration of the key parameters, resulting in a second-order polynomial model that exhibited a strong predictive accuracy (R^2^ = 0.958) and statistical validation further confirming its reliability. Furthermore, the optimal removal conditions produced an experimental removal percentage (∼95.21–97.9%) closely matching the predicted value (∼97.41–98.0%), demonstrating the robustness of the RSM-based optimization strategy. Kinetic modeling showed that MB adsorption followed a pseudo-second-order mechanism, indicative of chemisorption driven by valence forces or electron exchange, with equilibrium data aligned best with the Freundlich isotherm model (R^2^ = 0.972), reflecting heterogeneous surface characteristics and multilayer adsorption. Thermodynamic analyses verified that MB uptake was endothermic and spontaneous, with negative Gibbs free energy values and increased adsorption favorability at higher temperatures. The synthesized mt-NADES biopolymer beads exhibited better reusability, maintaining >95% removal efficiency over six consecutive adsorption–desorption cycles.

Overall, this work demonstrates a green, efficient approach that integrates solvent chemistry, biopolymer engineering, and statistical optimization to produce environmentally friendly, thermally stable, and pH-responsive adsorbent beads. The performance and reusability of the mt-NADES-encapsulated alginate-chitosan biopolymer beads highlight their potential for wastewater treatment and related applications. Nevertheless, the absence of studies evaluating the effect of coexisting salts and interfering ions represents a limitation of the current work, and future investigations in complex aqueous systems will be essential to establishing adsorption selectivity and real-world performance.

## Supplementary Material



## Notes

Materials and chemicals; instrumentation; schematic of mt-NADES synthesis; NMR, LCMS-ESI, and physicochemical properties of menthol, thymol, and mt-NADES data; point of zero charge for mt-NADES beads; CCD variables and responses for the removal of MB; studentized residual data; model evaluation comparing the predicted and experimental removal percentages of MB; Freundlich, Langmuir, and Temkin parameters for MB adsorption; kinetics parameters for the pseudo-first- and pseudo-second-order; thermodynamic parameters for MB adsorption on mt-NADES beads; FTIR spectra of mt-NADES beads before and after the reuse cycles; comparative analysis for various desorbing agents and reusability comparison with literature (PDF)
