# Supplementary material for: Development of mt-NADES Beads and Optimization for Efficient Extraction of Methylene Blue Using Response Surface Method
Source: ACS Omega. 2026 May 26;11(22):32483–98. doi: 10.1021/acsomega.6c00978 (PMC13261482; doi:10.1021/acsomega.6c00978)
Supplement: Supplementary file 1 [file ao6c00978_si_001.pdf]

Supporting information

Development of mt-NADES Beads and Optimization for Efficient Extraction of Methylene Blue Using Response Surface Method

*Taiwo Bakare-Abidola, Seth K. Smerjac, Kyle Jorgensen, William J.A. Russell, Rahul Sampat Khandge, and Rocío L. Pérez_**

Center for Advanced Materials Science (CAMS); Department of Biochemistry, Chemistry, and Physics; Georgia Southern University, Statesboro, GA 30458.

*Corresponding author: rperez@georgiasouthern.edu

No of Scheme: 1
No of Figures: 5
No of Tables: 9

1. Experimental Section

1.1 Materials and Chemicals

Methylene blue dye (MB) (≥97.0%, 319.85g/mol), Thymol ($C_{10}H_{14}O$, 150.22 g/mol, ≥98.5%), Chitosan (≥75% deacetylation degree), and Sodium Alginate (≥90.8%) were purchased from Thermo Fisher Scientific (*Nazareth, PA, USA*). DL-menthol ($C_{10}H_{20}O$, 156.27 g/mol, ≥95%), Ethanol (C_2H_5OH, ≥99.5%, 46.07 g/mol) and Hydrochloric acid (HCl, 36.46 g/mol, ≥37%) were procured from Sigma-Aldrich (USA). Calcium chloride ($CaCl_2$, ≥97%, 110.98 g/mol), Sodium Chloride (NaCl, ≥99.7%, 58.44 g/mol) was obtained from VWR Chemicals (USA). All chemicals were used as received without further purification.

1.2 Instrumentation

Fourier-transform infrared (FT-IR) spectra were obtained using Nicolet iS20 spectrometer (Thermo Scientific) collecting 256 scans for spectrum in the 500-4000 cm^{-1} wavelength range. ^{1}H and ^{13}C NMR spectra of NADES were acquired on a JEOL ECZL-400S spectrometer (400 MHz). Liquid chromatography with electrospray ionization mass spectrometry (LCMS-ESI) was performed to confirm the synthesis of NADES. Rheological properties were measured using a Brookfield DVNext Cone/Plate Rheometer (shear rate: 0.1–100 s^{-1}, temperature: 25–60 °C). Melting point measurements were conducted using a Mel-Temp II Digital Melting Point Apparatus (Laboratory Devices, model 1201D). Zeta potential of the beads was acquired using Malvern Zetasizer (Malvern Instruments, UK) with a 10 mM NaCl solution with a pH ranging from 3.0 ± 0.2 to 12.50 ± 0.2. Thermogravimetric analysis (TGA) was performed using a TA Q50 instrument (25–700 °C, 20 °C/min, N_2 atmosphere). Surface morphology of the samples was examined by field-emission scanning electron microscopy (FE-SEM, JEOL JSM-7600F). Ultraviolet-visible

(UV-Vis) spectra were collected using a Jasco V-750 spectrophotometer to monitor MB concentrations. The pH of the solutions was monitored with a Mettler Toledo pH meter. Deionized water (≥18.2 MΩ cm, Milli-Q system) was used throughout the study.

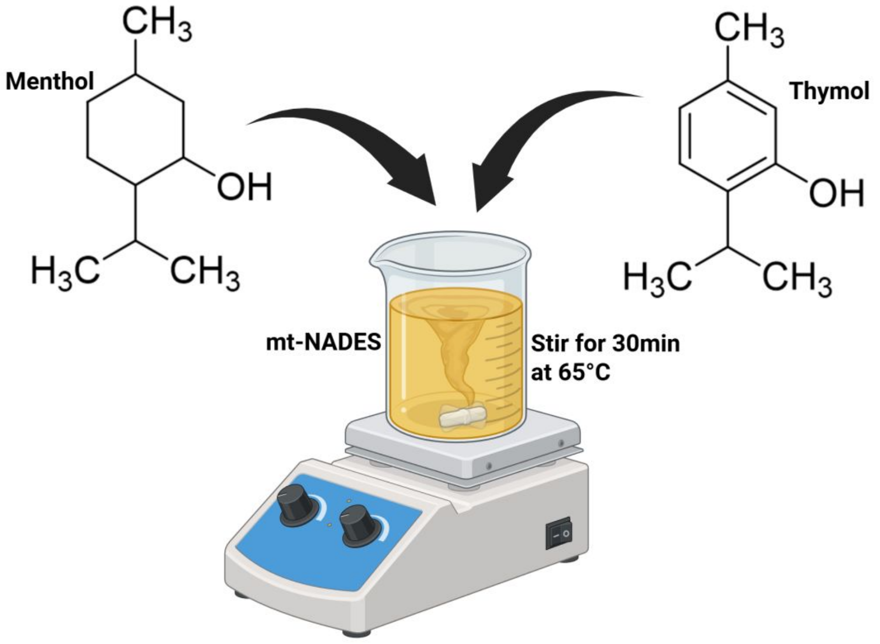

Scheme S1. Synthesis of mt-NADES

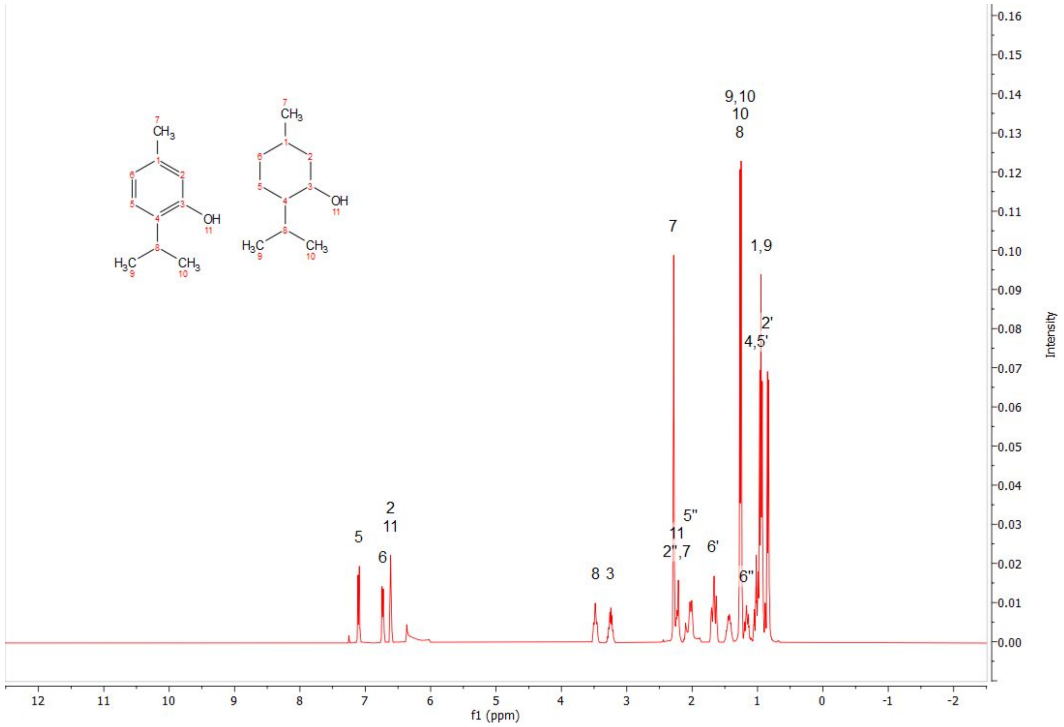

Figure S1: ^{1}H NMR (400 MHz, DMSO-D6) δ 8.99 (d, J = 1.6 Hz, 1H), 6.92 (d, J = 7.8 Hz, 1H), 6.60 (d, J = 2.0 Hz, 1H), 6.51 (dd, J = 7.8, 1.8 Hz, 1H), 4.32 (s, 1H), 3.23 – 3.12 (m, 2H), 2.22 (dddd, J = 13.9, 8.9, 6.8, 2.0 Hz, 1H), 2.16 (d, J = 1.4 Hz, 3H), 2.03 (s, 1H), 1.85 (ddq, J = 11.4, 4.1, 2.1 Hz, 1H), 1.54 (ddq, J = 28.6, 12.5, 3.0 Hz, 2H), 1.31 (dddt, J = 11.8, 9.9, 6.5, 3.4 Hz, 1H), 1.17 – 1.10 (m, 6H), 1.05 – 0.99 (m, 1H), 0.99 – 0.87 (m, 2H), 0.87 – 0.81 (m, 6H), 0.78 (dd, J = 12.0, 3.2 Hz, 1H), 0.74 (dd, J = 7.0, 1.6 Hz, 3H).

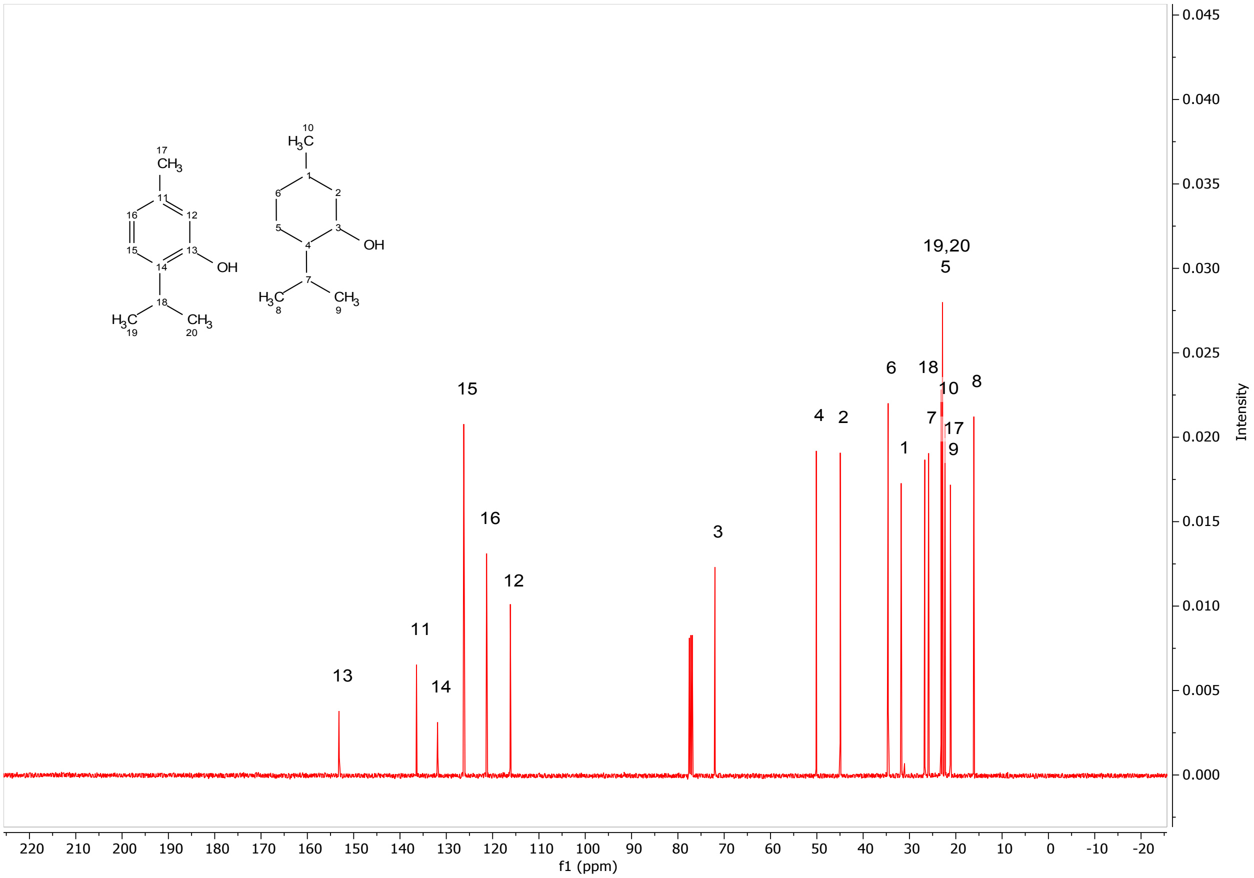

Figure S2: ^{13}C NMR (400 MHz, DMSO-D6) δ 153.17 (C13), 136.46 (C11),131.88 (C14), 126.23 (C15), 121.32 (C16), 121.30 (d, 6), 116.20 (C12), 72.00 (C3), 71.98, 50.12 (C4), 44.94 (C2), 34.59 (C6), 31.79 (C1), 26.70 (C18), 25.86 (d, 7), 23.17, 22.87 (t, C5, C19, C20), 22.33 (C10), 21.17 (q, C9, C17), 16.11 (C8).

Table S1: ESI data from synthesized mt-NADES.

	Theoretical m/z	Experimental m/z
Thymol	150.22	150.50
Menthol	156.15	156.25

Table S2: Physicochemical Properties of Menthol, Thymol, and mt-NADES.

Compound	Melting Point (°C)	Density (g/cm³)	Viscosity (mPa·s)
Menthol	42–45	0.890	~6–8 mPa·s at ~45 °C
Thymol	49–51	~0.96–0.97	~8–12 mPa·s at ~50–60 °C
mt-NADES	31-32	~0.91–0.97	~27.5–53

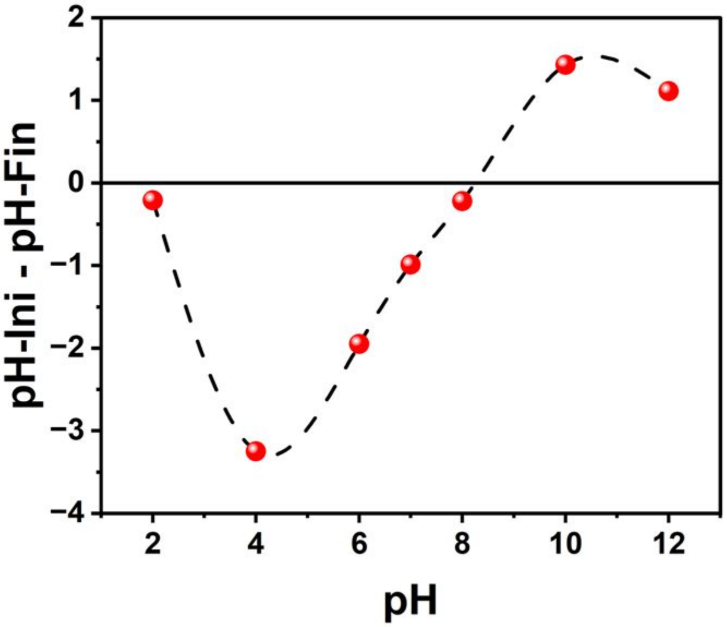

Figure S3: Point of zero charge plot for the mt-NADES beads

Table S3: Variables and responses of the CCD carried out for the removal of MB.

Exp.	Mass (mg)	pH	[MB] (ppm)	Time (min)	R%
1	20	12	50	15	39 (+/- 1)
2	200	12	500	15	43 (+/- 2)
3	20	2	50	15	5 (+/- 2)
4	20	2	50	240	38 (+/- 3)
5	110	7	500	127.5	87 (+/- 2)
6	200	12	50	240	98 (+/- 2)
7	110	7	50	127.5	88 (+/- 4)
8	110	7	275	240	93.8 (+/- 0.4)
9	200	12	500	240	103.1 (+/- 0.6)
10	110	2	275	127.5	50.4 (+/- 0.2)
11	200	2	50	240	62 (+/- 1)
12	20	12	500	15	11 (+/- 2)
13	110	7	275	15	56 (+/- 2)
14	20	2	500	240	9.8 (+/- 0.6)
15	110	7	275	127.5	90.2 (+/- 0.5)
16	200	7	275	127.5	90.25 (+/- 0.03)
17	110	12	275	127.5	86 (+/- 3)
18	20	12	50	240	97 (+/- 1)
19	20	2	500	15	6 (+/- 1)
20	200	2	500	15	24 (+/- 3)
21	200	2	500	240	82 (+/- 2)
22	20	12	500	240	69 (+/- 5)
23	20	7	275	127.5	52 (+/- 2)
24	110	7	275	127.5	65.53 (+/- 0.05)
25	200	12	50	15	86 (+/- 2)
26	200	2	50	15	34 (+/- 4)

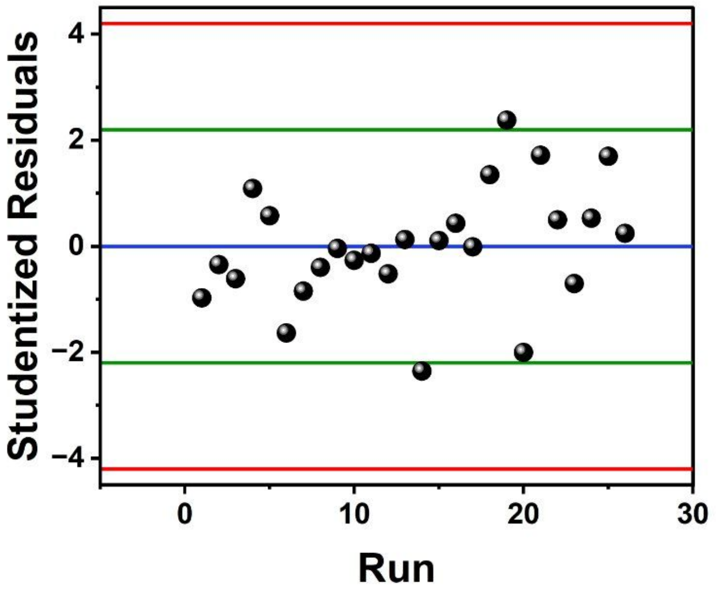

Figure S4: Studentized residuals versus the runs of the experimental design.

Table S4: Model evaluation comparing the predicted and experimental removal percentages of MB under different experimental and optimal conditions using Chit-Alg Beads and mt-NADES Bead.

Exp.	Mass	pH	Time	[MB]	R% predicted	Chit-Alg Beads R%	mt-NADES Beads R%	E (%)
1	50	10	150	275	67.02	45.5 (+/- 0.8)	67.3 (+/- 0.4)	+21.8
2	200	6	195	140	93.33	56.90 (+/-1.36)	91.4 (+/- 0.7)	+34.5
3	150	2	200	300	64.32	37.37 (+/-0.84)	67 (+/- 2)	+29.6
4	100	7	200	100	97.95	56.56 (+/-2.93)	91.6 (+/- 0.2)	+35.0
5	155	8	150	275	96.20	53.66 (+/-0.69)	94.5 (+/- 0.3)	+40.8
6	75	5	150	100	68.20	36.56 (+/-2.35)	62.4 (+/- 0.5)	+25.8
**Optimal**	155	10	150	275	97.41	55.91 (+/- 0.43)	95.2 (+/- 0.4)	+39.3

* Mass (mg); Time (min); [MB] (ppm); E = Enhancement (%); Chit-Alg: Chitosan-Alginate

Table S5: Freundlich, Langmuir and Temkin parameters for the adsorption of MB on the mt-NADES beads.

Model	Parameters	Value
Freundlich	Kf	916.64
	n	1.62
	R^2	0.9717
	SSE	2.16×10^{-2}
	MSE	2.71×10^{-3}
	χ^2	8.30×10^{-3}
Langmuir	q max	769.23
	KL	4.333
	R^2	0.8931
	SSE	4.52×10^{-5}
	MSE	4.52×10^{-6}
	χ^2	5.88×10^{-3}
Temkin	B1	490.55
	Kt	37.92
	R^2	0.8118
	SSE	9.91×10^{4}
	MSE	12.39×10^{3}
	χ^2	2.86×10^{2}

Table S6: Kinetics parameters for the pseudo-first- and pseudo-second order

Model	Parameters	Value
Pseudo-first order	Q_e	1004.25
	K1	0.0056
	R^2	0.8753
	SSE	0.2125
	MSE	0.019319574
	χ^2	0.03276
Pseudo-second order	Q_e	181.81
	k2	3.025×10^{-5}
	R^2	0.9971
	SSE	4.52×10^{-5}
	MSE	4.52×10^{-6}
	χ^2	5.88×10^{-3}

Table S7: Thermodynamic parameters for MB adsorption on mt-NADES beads

Temperature (°K)	ΔG (kJ/mol)	ΔH (kJ/mol)	ΔS (J/molK)
310	-12.50	125.32	-174.97
316	-13.68		
322	-14.37		
328	-15.04		
334	-17.33		
340	-17.83		
346	-18.94		
352	-19.52		

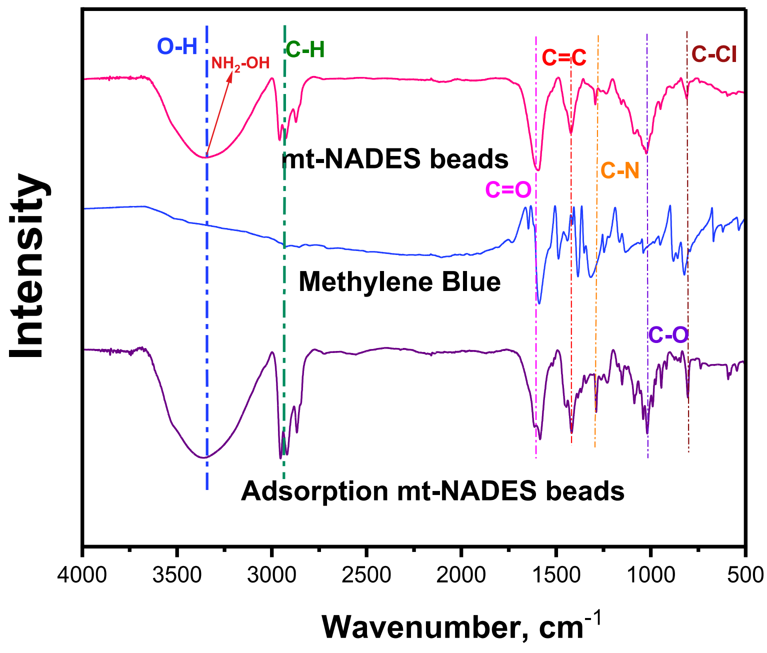

Figure S5: FTIR spectra of mt-NADES beads before and after the methylene blue dye adsorption

2. Comparison with Other Desorbing Agents

A comparative study with other desorbing agents (*Table S8*) confirmed that NaCl alone is ineffective, with removal efficiency dropping from 51.6% in cycle 1 to 24.1% in cycle 2. This

dramatic decrease demonstrates that without ethanol, NaCl cannot effectively desorb MB from the adsorbent, most likely due to: (i) Lack of MB solubility enhancement (ii) Poor penetration into hydrophobic NADES regions, (iii) Higher surface tension limiting pore access, however, further studies would confirm this claims.

In contrast, NaOH alone maintained 83-86% removal over six cycles, while HCl alone showed poor performance (36-59%). These comparisons validate the superior performance of our NaCl-ethanol system and justify its selection as the desorption medium. The presence of ethanol is critical for achieving complete regeneration, as it might addresses both solubility and penetration limitations of NaCl alone.

Table S8: Comparison with Other Agents

Regeneration Method	Cycle 1 Removal (%)	Cycle 2 Removal (%)
0.5 M NaCl 50:50V/V (EtOH/H_2O) (this work)	96.9%	99.5%
NaOH only	83.2%	86.0%
HCl only	46.8%	59.4%
0.5 M NaCl (no EtOH)	51.6%	24.1%

3. Reusability Comparison with Literature

The reusability performance of mt-NADES/alginate-chitosan beads compares favorably with previously reported adsorbents (*Table S9*). Luong et. al., reported that GO/chitosan granules achieved 82.7% desorption efficiency with ethanol 70%, while our system maintained >95% removal over six cycles [1]. Rahmatpour and team, observed 78.5% removal after five cycles using 0.1 M HCl for xanthan gum-chitosan/GO hydrogels [2]. Kausar et. al., achieved 79% desorption

with HCl for cellulose/clay/alginate composites [3], and Majamo et. al., reported corncob cellulose hydrogel could be reused for three cycles with <10% efficiency loss using 0.1 M NaOH [4].

The superior reusability of our adsorbent could be hypothetically attributed to: (i) The optimized desorption medium that combines ion exchange (Na^+) with solubility enhancement and pore penetration (ethanol), effectively removing MB without damaging the biopolymer matrix. (ii) The stable biopolymer matrix (alginate-chitosan) that withstands multiple regeneration cycles (iii) The hydrophobic mt-NADES components that remain entrapped within the matrix without leaching during desorption.

Table S9: Comparison of reusability performance with literature

Adsorbent	Desorbing Agent	Cycles	Performance Retained	References
GO/Chitosan granules	Ethanol 70%	7	82.7% (cycle 1)	[1]
Xanthan gum-Chitosan/GO	0.1 M HCl	5	78.5%	[2]
Cellulose/Clay/Alginate	0.1 M HCl	Not specified	79%	[3]
Corncob cellulose hydrogel	0.1 M NaOH	3	~90%	[4]
**mt-NADES/Alginate-Chitosan**	**0.5 M NaCl + Ethanol**	**6**	**>95%**	This work

References

[1] H.V.T. Luong, T.P. Le, T.L.T. Le, H.G. Dang, T.B.Q. Tran, A graphene oxide based composite granule for methylene blue separation from aqueous solution: Adsorption, kinetics and thermodynamic studies, Heliyon 10 (2024). https://doi.org/10.1016/j.heliyon.2024.e28648

[2] A. Rahmatpour, A.H.A. Hesarsorkh, Self-assembly of graphene oxide containing bio-nanocomposite hydrogels for removal of chlorpyrifos and methylene blue, Int. J. Biol. Macromol. 320 (2025). https://doi.org/10.1016/j.ijbiomac.2025.145850

[3] A. Kausar, S.U. Rehman, F. Khalid, A. Bonilla-Petriciolet, D.I. Mendoza-Castillo, H.N. Bhatti, S.M. Ibrahim, M. Iqbal, Cellulose, clay and sodium alginate composites for the removal of methylene blue dye: Experimental and DFT studies, Int. J. Biol. Macromol. 209 (2022) 576–585. https://doi.org/10.1016/j.ijbiomac.2022.04.044

[4] S.L. Majamo, T.A. Amibo, D.T. Mekonnen, Experimental investigation on adsorption of methylene blue dye from wastewater using corncob cellulose-based hydrogel, Sci. Rep. 14 (2024). https://doi.org/10.1038/s41598-024-54511-0
